# A high-resolution transcriptome map identifies small RNA regulation of metabolism in the gut microbe *Bacteroides thetaiotaomicron*

**DOI:** 10.1038/s41467-020-17348-5

**Published:** 2020-07-16

**Authors:** Daniel Ryan, Laura Jenniches, Sarah Reichardt, Lars Barquist, Alexander J. Westermann

**Affiliations:** 1 Helmholtz Institute for RNA-based Infection Research (HIRI), Helmholtz Centre for Infection Research (HZI), Würzburg, Germany; 2 Faculty of Medicine, University of Würzburg, Würzburg, Germany; 3 Institute of Molecular Infection Biology (IMIB), University of Würzburg, Würzburg, Germany

**Keywords:** RNA, Bacterial genomics, Bacterial transcription

## Abstract

Bacteria of the genus *Bacteroides* are common members of the human intestinal microbiota and important degraders of polysaccharides in the gut. Among them, the species *Bacteroides thetaiotaomicron* has emerged as the model organism for functional microbiota research. Here, we use differential RNA sequencing (dRNA-seq) to generate a single-nucleotide resolution transcriptome map of *B*. *thetaiotaomicron* grown under defined laboratory conditions. An online browser, called ‘Theta-Base’ (www.helmholtz-hiri.de/en/datasets/bacteroides), is launched to interrogate the obtained gene expression data and annotations of ~4500 transcription start sites, untranslated regions, operon structures, and 269 noncoding RNA elements. Among the latter is GibS, a conserved, 145 nt-long small RNA that is highly expressed in the presence of *N*-acetyl-D-glucosamine as sole carbon source. We use computational predictions and experimental data to determine the secondary structure of GibS and identify its target genes. Our results indicate that sensing of *N*-acetyl-D-glucosamine induces GibS expression, which in turn modifies the transcript levels of metabolic enzymes.

## Abstract (web summary)

*Bacteroides thetaiotaomicron* is a human gut microbe and an emergent model organism. Here, Ryan et al. generate single-nucleotide resolution RNA-seq data for this bacterium and map transcription start sites and noncoding RNAs, one of which modulates expression of metabolic enzymes.

## Introduction

*Bacteroides* are Gram-negative, obligate anaerobic, non-motile, non-spore forming rods, and among the most abundant bacterial genera in the human intestine^1^. *Bacteroides thetaiotaomicron* has emerged as a model representative of the gut microbiota, due to its widespread distribution among human populations and the relative ease of studying these bacteria under laboratory conditions^1^. The extensive metabolic potential encoded in the *B*. *thetaiotaomicron* genome, including 88 defined polysaccharide utilization loci (PULs)^2,3^, forms the prerequisite for gut colonization. However, persistence within this notoriously dynamic niche requires coordinated control of gene expression in response to fluctuating nutrient levels.

Bacteroidetes lack the classical housekeeping sigma factor (σ^70^) encoded by the proteobacterial *rpoD* gene, and consequently, the classical –10 and –35 boxes recognized by σ^70^ are absent from their promoters. Rather, members of this phylum contain an unusual, RpoD-like primary transcription factor, σ^ABfr^, as well as an arsenal of alternative, extra-cytoplasmic function (ECF) sigma factors^4,5^. The consensus recognition sequence of σ^ABfr^ was deduced by manual inspection of 23 promoter sequences in *B*. *fragilis* as ‘TAnnTTTG’ at the –7 region and a ‘TTTG’ motif at around the –33 region^6,7^. Inspection of global RNA-seq data recently confirmed the –7 consensus in another Bacteroidetes member, *Flavobacterium johnsoniae*, whereas the –33 motif could not be identified^8^. While in *B*. *fragilis* the sequence motif recognized by the oxidative stress-related alternative ECF sigma-factor EcfO was recently identified^9^, no such motif is currently known for any of the *B*. *thetaiotaomicron* alternative ECFs. Besides sigma factors, SusR-like regulators^10^ and hybrid two-component signal transduction systems^11,12^, wherein sensor kinase and response regulator are fused into a single polypeptide, contribute further to the adaptation of *Bacteroides* gene expression upon sensing of defined environmental cues, and DNA recognition motifs have been predicted for some of these regulators based on comparative genomics^13,14^.

Knowledge of post-transcriptional control mechanisms is sparse in *B*. *thetaiotaomicron*. Lacking classical Shine-Dalgarno (SD) sequences, messenger RNAs (mRNAs) of Bacteroidetes were recently shown to be enriched for adenine residues at position –3, –6, and –11 to –15 relative to the translational start codon, with adenine overrepresentation at these positions positively correlating with translation efficiency^8^. In Proteobacteria, small regulatory RNAs (sRNAs) can regulate target mRNAs through imperfect base-pairing interactions that typically occlude the SD sequence and/or the start codon, thus interfering with translation initiation^15^. As of now, only two sRNAs are known in *Bacteroides* spp. RteR is a *trans*-encoded sRNA of 90 nt that promotes discoordinate expression of the *tra* operon, required to assemble the mating apparatus for the transfer of the conjugative transposon, CTnDot^16,17^. DonS is a representative of a family of 78–128 nt-long *cis*-antisense RNAs divergently encoded to—and negatively impacting expression of—*susC* homologs of PUL systems involved in the binding and degradation of mucosa-derived glycans^18^. Lastly, a mRNA leader sequence was recently shown to tie *B*. *thetaiotaomicron* colonization to the presence of dietary sugars—and the authors speculated an sRNA could be involved in this process^19^. Together, these examples imply that RNA-mediated control mechanisms may be commonly employed by *B*. *thetaiotaomicron* to couple expression of metabolic genes to nutrient availability. However, the mode-of-action for all of these regulators is currently unknown, and RNA biology in *B*. *thetaiotaomicron* has not yet been investigated in a systematic manner. Particularly, given the lack of SD sequences in Bacteroidetes mRNAs, it is an open question if and how *trans*-encoded sRNAs could post-transcriptionally regulate target transcripts in this phylum.

Here, we performed differential RNA sequencing (dRNA-seq)^20,21^ of *B*. *thetaiotaomicron* grown in rich medium in three defined growth phases. This led to the annotation of a total of 4507 transcription start sites (TSSs) and untranslated regions (UTRs), as well as the identification of promoter motifs, RNA processing sites, and operon structures. To provide easy access to our transcriptome data, we have developed the intuitive Open-Access online database ‘Theta-Base’. We report the identification of 269 noncoding RNA candidates from all major classes including *cis-* and *trans-*encoded, 5′- and 3′-derived, and intra-operonic sRNAs as well as riboswitches, RNA thermometers, and putative type-I toxin–antitoxin (TA) systems. We selected one of the newly identified intergenic sRNAs (GibS), determined its secondary structure, and—as the first example of a *trans*-encoded Bacteroidetes noncoding RNA—identified its target mRNAs in a genome-wide screen. Biochemical and genetic experiments revealed that GibS utilizes a highly conserved, single-stranded seed sequence in its 5′ portion to mediate base-pairing with the translation initiation regions of two metabolic target mRNAs (*BT_0771*, *BT_3893*), resulting in repression of the respective transcripts. Altogether, the presented data imply that *B*. *thetaiotaomicron* employs riboregulatory mechanisms for adapting its gene expression to changing environmental conditions and should foster future studies to explore RNA biology in bacterial members of the human microbiota.

## Results

### TSSs of *B. thetaiotaomicron* VPI-5482 grown in rich medium

**Fig. 1:**
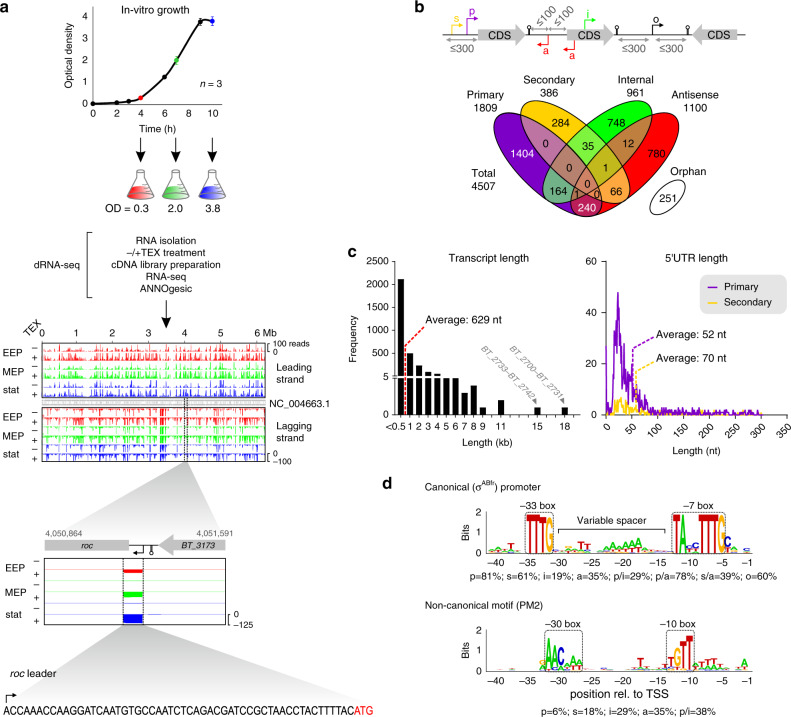
High-resolution view at the *B*. *thetaiotaomicron* transcriptome. **a** Experimental workflow of the dRNA-seq approach and close-up view on the transcription start site (TSS; indicated by a bent arrow) of the *regulator of colonization* (*roc*) gene. Bacteria (AWS-001) from three different growth phases in TYG medium—early exponential (EEP), mid-exponential (MEP), and stationary phase (stat)—were analyzed. TEX, terminator exonuclease. Growth data refer to the mean +/− standard deviation from three biological replicates. For the read coverage plots, one representative replicate out of three is depicted. **b** Definition of categories for TSS annotation (upper) and overlap among TSS categories (lower). CDS, coding sequence. Note that pTSS and sTSS are mutually exclusive (thus no overlap in the Venn diagram). **c** Length distribution of transcripts (left) and 5′ UTRs (right). Long operons are labeled by name. **d** Motif searches upstream of *B*. *thetaiotaomicron* TSSs reveal the canonical σ^ABfr^ promoter (upper) and a second, near-palindromic motif (‘PM2’; lower). Percentage values below the respective motif denote the frequency of that sequence upstream of the respective TSS category as defined in **b**. Source data to this figure are provided in the Source Data file.

By selectively enriching triphosphates at the 5′ end of primary transcripts, dRNA-seq identifies TSSs in a genome-wide manner, resulting in high-resolution transcriptome maps^20,21^. To globally identify TSSs in *B*. *thetaiotaomicron*, we transferred the dRNA-seq protocol to total RNA samples extracted from the type strain *B*. *thetaiotaomicron* VPI-5482 in three defined growth phases in TYG (tryptone-yeast extract glucose) medium—at the transition from lag to exponential phase, in mid-exponential and stationary phase—and analyzed the resulting data using the ANNOgesic bioinformatic tool set^22^ (Fig. 1a). This approach reliably mapped TSSs in the *B*. *thetaiotaomicron* genome, as illustrated for the *roc* (*regulator of colonization*) mRNA for which dRNA-seq identified the exact TSS as previously mapped using primer-extension^19^.

In the *B*. *thetaiotaomicron* core genome and plasmid, we identified a total of 4507 TSSs that were classified into five categories based on their genomic location relative to annotated CDSs (Fig. 1b). Primary TSSs (pTSSs; 40% of all TSSs) were defined as the TSS with the highest coverage and secondary TSSs (sTSSs; 8%) as all remaining TSSs within a 300 nt window upstream of a given CDS. Internal TSSs (iTSSs; 21%) and antisense TSSs (aTSSs; 24%) arise from within a given CDS (or in case of an aTSS, within a 100 nt window flanking the CDS), in either sense or antisense orientation, respectively. The remaining 5% of predicted TSSs, not associated with any CDS, were classified as orphan (oTSSs) and might reflect an incomplete annotation of open reading frames or be indicative of intergenic sRNA genes (see below). Of the predicted TSSs, 1951 were detected in all growth phases with the total number of TSSs detected per condition ranging between 2265 and 3606 (Supplementary Data 1). The majority of TSSs were detected in stationary phase (~34% expressed exclusively under this condition; Supplementary Data 1). Functional analysis of genes with stationary phase-specific pTSSs using the PANTHER Classification System^23^ revealed enrichment of gene ontology (GO)-terms including DNA recombination, DNA integration, and integral membrane components (Supplementary Data 1).

Antisense transcription is widespread in bacteria, with between 5 and 75% of CDSs exhibiting antisense transcription depending on bacterial species, experimental condition, and RNA-seq protocol^24^. While functions for a number of specific antisense transcripts have been described^25^, their relevance as a general functional class remains unclear. Manual inspection of the predicted aTSSs in *B*. *thetaiotaomicron* revealed ~22% of them to serve as pTSS for CDSs further downstream (i.e., exceeding the set window of 300 nt; Fig. 1b), whereas the majority (73%) of identified aTSSs gave rise to relatively short, weakly expressed transcripts, suggesting some aTSSs may arise from spurious transcription initiation rather than being associated with a functional transcript^26^. For future analyses of specific antisense transcripts in *B*. *thetaiotaomicron* we advise readers to consider the here-reported aTSSs and consult the provided database (which will be described below). Manual inspection of predicted iTSSs revealed several examples of transcriptionally inter-connected genes/operons with related functions, e.g., the TSS for *BT_3336*, involved in the biosynthesis of lipid A, is located within the upstream *oprM* gene for an efflux pump involved in multidrug resistance, highlighting the link between cell envelope modifications and antibiotic stress.

Sequence alignment of all identified TSSs revealed the purine bases adenine and guanine as the preferred initiating nucleotides (45% or 41% of cases, respectively). As purine triphosphates serve as major energy storage molecules in cells, purine base overrepresentation in initiator nucleotides may help couple transcriptional activity to metabolic state, as observed previously in aerobic bacteria^27–29^.

### Global assessment of transcript features

By combining TranstermHP^30^ and RNAfold^31^, ANNOgesic predicts intrinsic transcription terminators, which—together with the experimentally mapped TSSs—enables the deduction of transcript boundaries. *B. thetaiotaomicron* expressed transcripts in a range from 20 nt to ~18 kb (Fig. 1c, left). The longest transcripts correspond to ribosomal operons (*BT_2700*–*BT_2731* and *BT_2733*–*BT_2742*), cell surface and iron transport operons (*BT_1950*–*BT_1958*), and metabolic gene clusters (*BT_1099*–*BT_1108*).

5′ UTRs were inferred from the identified TSSs and annotated CDSs, revealing average and median lengths of 52 and 32 nt, respectively (Fig. 1c, right). Most mRNAs harbored a 5′ UTR of 23 nt; ~10 nt shorter than the assumed optimal length for translation initiation in Proteobacteria^20,32^. In line with leaderless mRNAs being considered rare in Gram-negative species^33^, our dRNA-seq screen identified only ~3.7% *Bacteroides* 5′ UTRs shorter than 10 nt, e.g. of mRNAs for several transposases (*BT_0280*, *BT_1996*), two-component sensor kinases (*BT_3967*, *BT_2166*), and transporters (*BT_0161*, *BT_0158*). In contrast, 13.5% of mRNAs had unusually long (>100 nt) 5′ UTRs that might serve as targeting platforms for sRNAs or RNA-binding proteins (RBPs), or contain *cis*-regulatory RNA elements. Indeed, as inferred from homology to known RNA elements from other bacteria, ~2% of the long 5′ UTRs were predicted to harbor a putative riboswitch (Supplementary Data 2). Manual inspection revealed yet other long 5′ UTRs to actually contain short open reading frames (sORFs; Supplementary Data 3) reminiscent of leader peptides mediating transcription attenuation^34^. For instance, the 5′ UTR associated with the mRNA for the hypothetical protein BT_4401 (462 nt in length) contains several putative sORFs (sORF_378 to −381), whose existence needs to be validated in the future.

Small proteins have long gone unnoticed, due to difficulties in annotation and detection, but recently this class of molecules has gained attention as biological functions for several representatives could be demonstrated^35,36^. In total, ANNOgesic predicted 409 sORF candidates in *B*. *thetaiotaomicron* over growth in TYG medium (Supplementary Data 3). However, only seven of those are supported by a recent ribosome profiling study^37^ (Supplementary Fig. 1a). To some extent, this divergence can be explained by the Ribo-seq study considering only conserved sORFs, thus deliberately accepting false-negatives for the sake of enriching true-positives. sORF candidates called exclusively in our screen might thus be strain-specifically encoded, condition-specifically expressed (growth for maximally 10 h in TYG in the present study vs. 72 h growth in brain infusion medium in ref. ^37^), or false-positives. Inspection of the Ribo-seq-exclusive sORFs revealed 18 of the 21 remaining candidates to possess few to no aligned RNA-seq reads in our experimental conditions, explaining their neglection in the present screen. The other three sORF candidates (NC_004663.1_3066620_3066739_−1, NC_004663.1_951512_951613_−1 and NC_004663.1_4594728_4594793_1 in ref. ^37^) were relatively highly expressed in our RNA-seq data, but located within annotated CDSs for larger proteins and, consequently, were not called as sORFs by ANNOgesic.

### *B. thetaiotaomicron* promoter architectures

A search for promoters upstream (–50 to +1) of the cognate TSS using the MEME^38^ and GLAM2^39^ toolkits identified two conserved motifs (Fig. 1d). Motif 1, consisting of two sequence elements centered at the –7 and –33 nt positions separated by an AT-rich spacer of variable length, strongly resembles the canonical σ^ABfr^ promoter of *B*. *fragilis*^6^. In *B. thetaiotaomicron*, this promoter was found upstream of 81% of pTSSs as well as 60% of sTSSs and oTSSs (Fig. 1d).

The second motif comprised sequence elements centered at the –10 and –30 positions relative to the TSS and was generally associated with more lowly expressed genes (Supplementary Fig. 1b). As is exemplified for *BT_4614* (Supplementary Fig. 1c), we noticed certain cases where transcription of genes driven by promoter motif 2 (PM2) initiated from a different TSS in stationary phase compared to earlier growth stages. More generally, functional annotation of all 75 PM2-containing coding genes in our study revealed an enrichment of the oxidative stress response (Supplementary Fig. 1d) and differential expression analysis an upregulation of oxidative stress-related genes in the stationary growth phase (Supplementary Fig. 1e).

### Theta-Base: an interactive online browser to interrogate the *Bacteroides* transcriptome

**Fig. 2:**
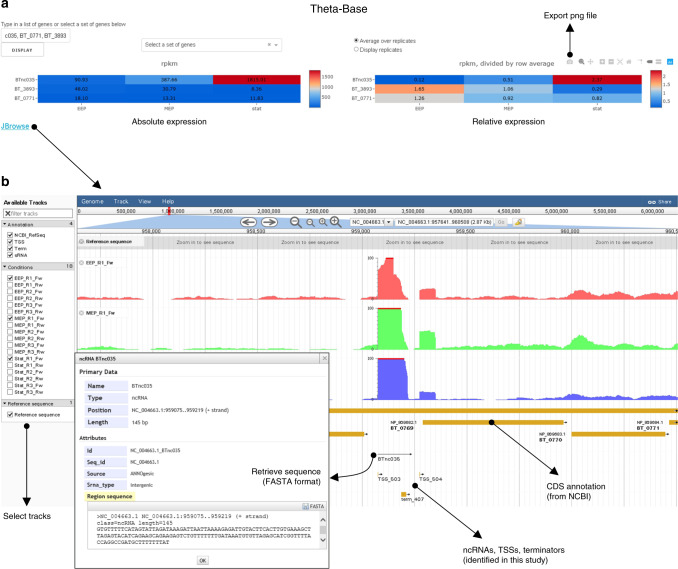
Theta-Base allows easy access to *B*. *thetaiotaomicron* transcriptomic features. **a** Heatmap representation of absolute (reads per kilobase of transcript per million mapped reads [rpkm]; left) or relative (rpkm divided by row average; right) transcript levels illustrates the anti-correlation in expression of GibS (a.k.a. BTnc035) and its repressed targets (*BT_0771*, *BT_3893*; as identified in Fig. 5) over growth in TYG. For visualization, query genes (coding and noncoding) may either be entered individually in the upper left box or selected from pre-defined gene sets. Biological replicates can be displayed individually or (as in the given example) the average expression over each three replicates may be shown. ‘EEP’ refers to early, ‘MEP’ to mid-exponential, and ‘stat’ to stationary phase. The heatmap can be exported as a graphic file in the upper right corner. At the lower left corner is a link to the genome browser (**b**). **b** JBrowse view on the read coverages over the GibS-encoding locus within the *B*. *thetaiotaomicron* genome. Tracks for display can be selected on the left (i.e., annotated coding and noncoding genes and transcript features [transcription start sites, terminators] as well as different experimental conditions and replicates). At the top, gene names or genome coordinates may be entered; zoom-in and -out functions exist. Inset: By clicking on any of the annotated features, primary sequences can be retrieved in FASTA format.

*B*. *thetaiotaomicron* is emerging as a model anaerobic bacterium; however, the community currently lacks an online repository for transcriptomic features and gene expression profiles. Inspired by online community data visualization platforms^40^ such as the SalCom^41^ and AcinetoCom^29^ databases compiled by the Hinton group for the bacterial pathogens *Salmonella enterica* and *Acinetobacter baumannii*, we generated Theta-Base as an intuitive online tool to easily interrogate the here-presented transcriptomic data. Theta-Base enables search queries for any annotated *B*. *thetaiotaomicron* coding gene as well as the here-identified noncoding genes (see below) and visualizes their expression profiles over growth in TYG in a simple heatmap format (Fig. 2a) that can be exported to the interactive graphing environment Plotly^42^. Moreover, a linkout to JBrowse^43^ allows the transcriptomic data to be viewed in the context of the *B*. *thetaiotaomicron* chromosome or plasmid (Fig. 2b), and additionally features the annotations of transcriptomic parts including TSSs and Rho-independent terminators. We hope this database will be routinely consulted by researchers of the *Bacteroides* community to retrieve information about transcript borders (relevant for cloning purposes or the design of PCR primers) or relative expression levels, and plan to further complement the current dataset with transcriptome profiles derived from *B*. *thetaiotaomicron* grown under various experimental conditions in the future. Theta-Base can freely be interrogated at www.helmholtz-hiri.de/en/datasets/bacteroides.

### The noncoding RNA landscape of *B*. *thetaiotaomicron*

**Fig. 3:**
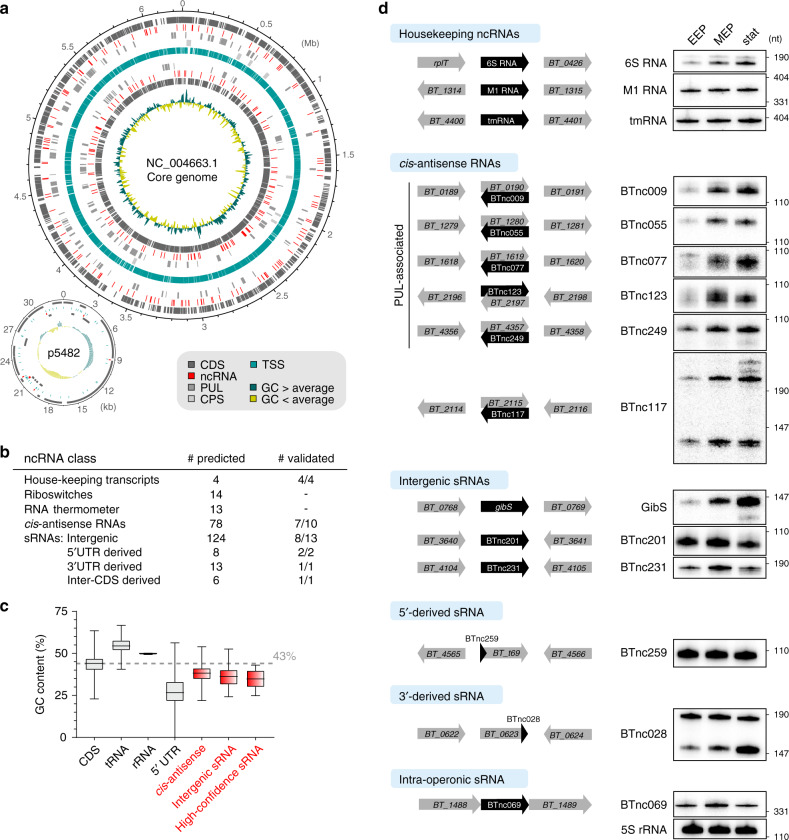
The noncoding transcriptome of *B*. *thetaiotaomicron*. **a** DNAPlotter maps^111^ denote the genomic position of the identified noncoding genes within the chromosome or plasmid, respectively. Position of coding sequences (CDS), polysaccharide utilization loci (PUL), and capsular polysaccharide synthesis loci (CPS) were retrieved from NCBI and published literature^5^. Positions of transcription start sites (TSS) and noncoding RNAs (ncRNA) were identified in the present study. **b** Overview table on the numbers of predicted and experimentally validated (probed/detected on northern blot) ncRNA candidates of the different classes. **c** GC content of different transcript classes. The center lines refer to the medians, the lower and upper borders of the boxes refer to the 25% and 75%, respectively, and whiskers indicate the minimal/maximal GC contents of CDSs (*n* = 4816), tRNAs (*n* = 71), rRNAs (*n* = 15), 5′ UTRs (*n* = 1373), *cis*-antisense RNAs (*n* = 78), and all putative or the high-confidence intergenic sRNAs (*n* = 124; 49). The dashed line represents the average GC content of the genome. **d** Northern blot validation of predicted ncRNA candidates. Left: sRNA locus orientation. Right: Total RNA was extracted from wild-type *B*. *thetaiotaomicron* (AWS-001) grown in TYG to early exponential (EEP), mid-exponential (MEP) or stationary phase (stat) and used for Northern blotting (representative images from each two biological replicates are shown). Apparent sizes in nucleotides are given to the right of the blot. 5S rRNA served as loading control. Source data to this figure are provided in the Source Data file.

Our dRNA-seq analysis uncovered a wealth of noncoding RNAs in *B. thetaiotaomicron* scattered throughout the genome (Fig. 3a). This included abundant housekeeping transcripts such as the RNA component of RNase P (M1 RNA) and transfer-messenger RNA (tmRNA). We also detected the *Bacteroides* 6S RNA and obtained evidence for the existence of product RNAs (pRNAs) (Supplementary Fig. 2a), which were previously predicted in the Bacteroidetes phylum^44^, but remained to be validated. In fact, we detected two classes of pRNAs in *B*. *thetaiotaomicron* (denoted pRNA and pRNA* in Supplementary Fig. 2a), reminiscent of previous observations in the ε-proteobacterium *Helicobacter pylori*^20^.

Noncoding RNA candidates were identified and classified on the basis of homology to described representatives of *cis*-regulatory elements into riboswitches and RNA thermometers (Rfam database^45^) or based on their genomic location into *cis*-antisense, intergenic, 5′- or 3′-derived and intra-operonic sRNAs (Fig. 3b; Supplementary Fig. 2b–i; Supplementary Data 2). ANNOgesic rediscovered three putative thiamine pyrophosphate (TPP) riboswitches^46,47^ in the 5′ UTRs of *BT_0653* (encoding the thiamine biosynthesis protein ThiS), *BT_2390* (for an outer membrane thiamine transporter), and *BT_2396* (for a protein involved in nicotinamide mononucleotide transport). Further, *B. thetaiotaomicron* expressed 78 *cis*-antisense and 124 intergenic sRNAs, respectively, during growth in rich medium; the latter number was reduced to 49 when requiring the presence of a Rho-independent terminator structure at the 3′ end as an additional selection criterion (along with a cognate TSS and a minimum read coverage; see Methods for details). We refer to this subset of 49 candidates as “high-confidence” intergenic sRNAs. Conservational analysis revealed 22 of these sRNAs to be conserved within two or more genera within the Bacteroidetes phylum, while the remaining 27 are strain-specific (Supplementary Fig. 3a). Analysis of the nucleobase composition of *cis*-antisense and intergenic sRNAs revealed GC contents of 38% or 35%, respectively, which is lower than that of CDSs, transfer RNAs (tRNAs) and ribosomal RNAs (rRNAs), in line with observations previously made in *Escherichia coli*^48^ (Fig. 3c). Selected *cis*-antisense RNA representatives were predicted to fold into extensive secondary structures, whereas several intergenic sRNAs exhibited extended unfolded regions (Supplementary Fig. 3b). Promoter analysis revealed the canonical σ^ABfr^ promoter to be associated with 52% or 76%, and the non-canonical PM2 with 24% or 13% of *cis*-antisense or intergenic sRNA candidates, respectively (Supplementary Data 2).

CRISPR (clustered regularly interspaced short palindromic repeats) systems protect their host against bacteriophage infections and are encoded by ~45% of sequenced bacterial genomes, including that of *B*. *fragilis*^49^. In contrast, no CRISPR array nor any Cas (CRISPR-associated) proteins have been annotated in *B*. *thetaiotaomicron*. In agreement with this, the CRISPR Recognition Tool^50^, which is included in the ANNOgesic pipeline, failed to identify any CRISPR-RNAs in our dataset.

### Experimental validation of *B*. *thetaiotaomicron* sRNAs

To validate the predicted sRNAs, we performed Northern blot assays for 31 randomly selected RNA candidates from diverse classes (Fig. 3d). DonS, the prototypical PUL-associated antisense RNA^18^, was not expressed when *B*. *thetaiotaomicron* grew in TYG medium and consequently, not identified in our screen. However, our transcriptomic approach readily detected ten further *cis*-antisense RNAs with a DonS-like genomic orientation, and we validated the expression of five of them (BTnc009, −055, −077, −249, and −123) by Northern blot (Fig. 3d). Of note, we observed anti-correlation in the expression of some of these antisense RNAs and their cognate *susC* homologs in our RNA-seq data (highlighted in red in Supplementary Fig. 4a), arguing that *B*. *thetaiotaomicron cis*-antisense RNAs may repress their corresponding PUL system in the presence of a prioritized carbon source, similarly to DonS in *B*. *fragilis*. Another seven pairs of *cis*-encoded antisense RNAs had a genomic architecture reminiscent of type-I TA systems, with each one of the divergently encoded genes harboring a candidate sORF. These putative TA systems showed a similar expression pattern with the presumed toxin mRNAs expressed at constant—albeit low—levels and the putative antitoxin RNA specifically induced in stationary phase (Supplementary Fig. 4b).

To assess the reliability of intergenic sRNA predictions, we independently tested the existence of selected candidates by Northern blot. This way, we validated nine of eleven tested candidates, from which eight (including BTnc035 [renamed to GibS: see below], BTnc201, and BTnc231; Fig. 3d; Supplementary Fig. 2d–f) possess both a TSS and an intrinsic terminator—features characteristic of canonical intergenic sRNAs. Thus—while not a hard selection criterion in the default ANNOgesic pipeline—the presence of a 3′ terminator may enhance our confidence in sRNA predictions and we therefore prioritized these high-confidence intergenic sRNAs in the following.

We also uncovered several putative sRNAs originating from the 5′ or 3′ UTR of mRNAs of which we validated one representative candidate each (BTnc259, BTnc028; Supplementary Fig. 2g, h) by Northern blot (Fig. 3d). Finally, ANNOgesic classified sRNAs into a fifth group termed inter-CDS-derived (more appropriately, intra-operonic) sRNAs. These are similar to 5′ UTR-derived sRNAs, however, they may originate at either a TSS or a processing site and can end at a Rho-independent terminator or a processing site. We identified six members belonging to this class, and experimentally validated BTnc069 (Fig. 3d; Supplementary Fig. 2i).

### GibS—an intergenic sRNA conserved within *Bacteroides* spp

**Fig. 4:**
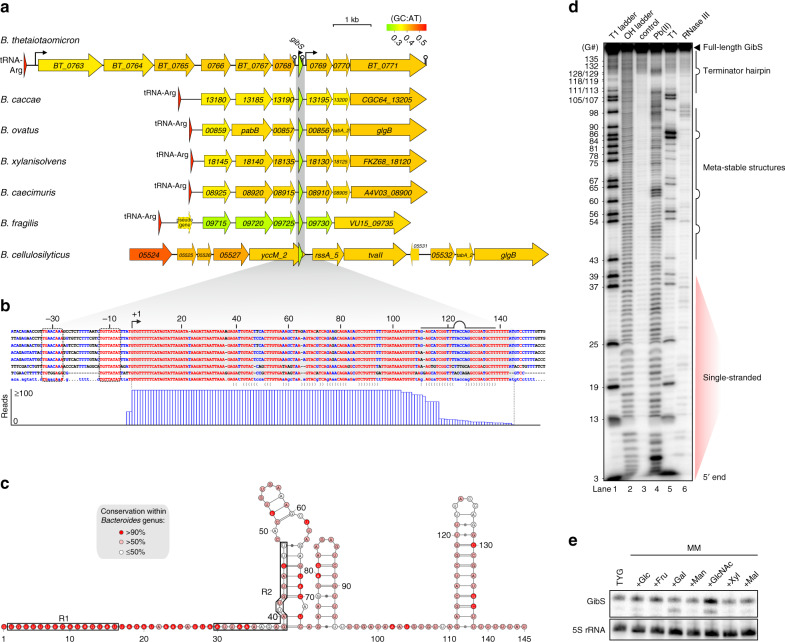
Conservation, secondary structure, and expression profile of GibS sRNA. **a** Shown is the overview of the *gibS* locus in seven indicated *Bacteroides* species. **b** Upper: Sequence alignment of the *gibS* gene and flanking regions in the same *Bacteroides* species as in **a**. The sequence at the bottom refers to the consensus and the brackets below indicate the determined intra-molecular base-pairings (see **c** and **d**). Red and blue colors indicate perfectly conserved and less-conserved ribonucleobases, respectively. The numbers denote the position relative to the 5′ end of GibS (+1 position; validated in Supplementary Fig. 5). Lower: Read coverage plot over the *gibS* locus (stationary phase, ‘–TEX’ sample). At the position of the presumed intrinsic terminator (indicated above the alignment) read coverage drops, likely due to strong hairpins being less efficiently converted into cDNA. **c** Secondary structure prediction of *B*. *thetaiotaomicron* GibS was performed using the webserver for aligning structural RNAs^52^ and reflects the consensus structure of 14 different prediction programs, considering both, minimal free energy and covariation. The colors of the ribobases reflect their conservation within *Bacteroides* and numbering is as in **b**. R1 and R2 seed regions as identified in Fig. 5e are labeled. **d** In vitro structure probing of 5′ end-labeled GibS confirms the in silico prediction (representative image from two independent replicate experiments is depicted). T1 and OH ladders refer to partial digestion under denaturing conditions with nuclease T1 (lane 1; cleaves unpaired G residues, indicated to the left), or alkali (lane 2; cleaves at all positions), respectively, and ‘control′ (lane 3) refers to untreated GibS. Lanes 4–6 reveal cleavages induced by lead (II) acetate (cleaves single-stranded nucleotides), RNase T1, or RNase III (cleaves double-stranded regions), respectively, under native conditions. **e** Northern blot-based expression profiling of GibS in wild-type *B*. *thetaiotaomicron* (AWS-001) grown to stationary phase in either TYG medium or in minimal medium (MM) in presence of glucose (Glc), fructose (Fru), galactose (Gal), mannose (Man), *N*-acetylglucosamine (GlcNAc), xylose (Xyl) or maltose (Mal), respectively, as the sole carbon source (representative image from two biological replicates). 5S rRNA was the loading control. Source data are provided in the Source Data file.

To successfully colonize and persist in the human gut, *B. thetaiotaomicron* relies in large part on its ability to rapidly adapt to the ever-changing conditions associated with this dynamic environment. Given that sRNAs in other organisms are known to regulate gene expression in response to specific environmental cues^15,51^, we wondered whether *B*. *thetaiotaomicron* would also harness its sRNA repertoire to adapt to environmental changes. Of the 49 high-confidence intergenic sRNAs identified, we focused on GibS (GlcNAc-induced *Bacteroides* sRNA; renamed from BTnc035 for reasons below)—a sRNA encoded in between a putative para-aminobenzoate synthase cluster (*BT_0763*–*68*) and a glycogen biosynthesis operon (*BT_0769*–*71*) (Fig. 4a). GibS was highly expressed during growth in TYG media, especially upon entry into the stationary growth phase (Fig. 3d). Moreover, its primary sequence and the non-canonical PM2 (–10 and –30 box; Fig. 1d) are well conserved within the *Bacteroides* genus (Fig. 4b, upper; Supplementary Fig. 3a); features indicative of a functional transcript.

Northern blotting indicated a ~145 nt-long RNA species to be the major GibS transcript form (Fig. 3d), in line with a potential terminator structure after which the read coverage in the RNA-seq experiment drops (Fig. 4b, lower). Primer-extension analysis confirmed the TSS of GibS as mapped by dRNA-seq (Supplementary Fig. 5). In silico RNA alignment and folding of GibS homologs using WAR (webserver for aligning structural RNAs^52^), which provides a consensus alignment and structure based on a range of methods that consider both minimal free energy and residue co-variation, indicated GibS to adopt a relatively unfolded conformation (Fig. 4c). Indeed, chemical and enzymatic in vitro probing largely confirmed this prediction, with the highly conserved 5′ sequence being mainly single-stranded (positions 1-38), the middle region forming two consecutive meta-stable hairpins, followed by another linear region and the Rho-independent terminator (Fig. 4d). Apart from the extended single-stranded 5′ region, this is reminiscent of the structure of well-characterized proteobacterial *trans*-acting sRNAs^53^.

Within its gut niche, *B*. *thetaiotaomicron* uses simple sugars as signals to control the expression of metabolic modules^12,54^. Given their ability to mediate rapid responses to metabolic stimuli^55^, sRNAs might be involved in the adaptation of *Bacteroides* gene expression to nutrient availability. To test if GibS exhibits a monosaccharide-specific expression pattern, we profiled its steady-state levels in *B. thetaiotaomicron* grown to stationary phase in minimal medium with defined simple sugars as the sole carbon source (Fig. 4e). While GibS expression showed considerable variation across the panel of carbon sources, growth in the presence of N-acetyl-D-glucosamine (GlcNAc) resulted in the strongest induction (~1.6 fold higher GibS levels in stationary phase in GlcNAc than in TYG). Thus, GibS is a conserved, largely unstructured sRNA in *B*. *thetaiotaomicron* that is induced in the presence of select monosaccharides.

### Identification of GibS targets

Toward characterizing the role of GibS in *B*. *thetaiotaomicron* physiology, we constructed a sRNA deletion mutant (∆*gibS*) by removing the *gibS* sequence from the chromosome. In addition, a *trans*-complementation strain (*gibS*+) was generated for which the *gibS* gene under control of a modified P1T_DP_ promoter^56^ (designated P1T_D_) was re-inserted at an unrelated position into the ∆*gibS* chromosome. The promoter modification involved removal of the proximal *tetO2* operator to ensure transcription of the sRNA from its native TSS, albeit with the cost of leaky expression (see Methods). However, upon addition of anhydrotetracycline (aTC; 200 ng mL^−1^), the resulting strain expressed GibS to wild-type levels (Supplementary Fig. 6). Interfering with GibS expression in bacteria grown in defined minimal medium in presence of GlcNAc as the sole carbon source resulted in subtle growth variations (Supplementary Fig. 7a). In contrast, strains grew nearly identically in the presence of glucose (Supplementary Fig. 7a).

**Fig. 5:**
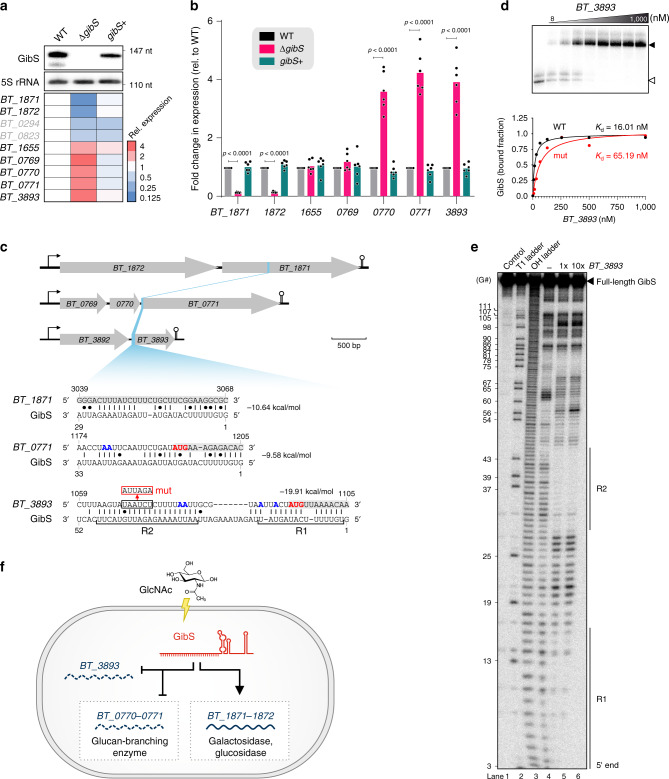
GibS regulates the expression of target genes likely via direct base-pairing. **a** Comparative transcriptomics of isogenic wild-type *B*. *thetaiotaomicron* (WT; AWS-003), a *gibS* deletion mutant (∆*gibS*; AWS-028), and complementation strain (*gibS*+; AWS-035). Upper: Northern blot showing stationary phase expression of GibS in the three strains. Heatmap below: RNA-seq data for putative targets under the same condition (i.e., fold change >2 or <0.5 at an FDR < 0.05 between WT and ∆*gibS*; mean of two biological replicates). Gray labeled genes were not considered further, as their expression pattern was not predictive of a GibS-specific effect. **b** qRT-PCR-based validation of the identified target candidates. 16S rRNA was used as a reference transcript. Bars denote the mean from three biological replicates each in technical duplicates (represented as single dots). Significant differences compared to wild-type expression levels were assessed using two-way ANOVA (Sidak’s multiple comparisons test; *p* values for significant [*p* < 0.05] comparisons are given). **c** Locus representation of target genes (upper) and their predicted interactions with GibS (lower). Red/blue nucleotides: start codon/Kozak-like sequences; gray shading: coding region; R1, R2: seed regions interacting with *BT_3893* (see **e**); ‘mut’: mutated nucleotides of *BT_3893* (for **d**). Minimal free energy values are to the right. Coordinates are relative to the TSS. **d** EMSAs support the predicted sRNA targeting site within *BT_3893*. T7-transcribed and 5′ end-labeled GibS was incubated with increasing concentrations of a ~150 nt-long 5′ segment of either wild-type (WT; black) or mutated variant of *BT_3893* (‘mut’; red; see **c**). *K*_d_ values represent the means of two independent replicate experiments. A representative gel from the WT is shown at the top and an image for binding to the mutated variant is given in Supplementary Fig. 8b (white and black arrowheads refer to free and bound GibS, respectively). **e** In-line probing of ∼0.2 pmol ^32^P-labeled GibS in the absence (lane 4) or presence of either 20 nM (lane 5) or 200 nM *BT_3893* mRNA leader (lane 6). Untreated RNA (lane 1; ‘control’), partially RNase T1- (lane 2; ‘T1’) or alkali-digested (lane 3; ‘OH’) GibS served as ladders. R1 and R2 denote GibS seed regions protected from cleavage in the presence of the target. Two independent replicate experiments were performed of which a representative image is depicted. **f** Working model for the role of GibS in *B*. *thetaiotaomicron*. Source data are provided as a Source Data file.

Next, we employed a genome-wide comparative transcriptomic approach to search for GibS-dependent expression changes. To this end, wild-type *B*. *thetaiotaomicron*, ∆*gibS*, and *gibS*+ strains were grown to stationary phase in TYG—i.e., a growth phase when endogenous GibS is highly expressed (Fig. 3d)—and their total RNA was extracted, depleted for rRNA, and sequenced. In the absence of GibS, four genes were repressed and five genes induced as compared to their wild-type expression levels (Fig. 5a). Notably, with the exceptions of *BT_0294* and *BT_0823*, expression of all these genes reverted to wild-type levels in the *trans*-complementation strain (Fig. 5a), indicating a sRNA-specific effect. *BT_1871*–*72*, i.e. the two genes seemingly activated by GibS (and thus, repressed in strain ∆*gibS*), comprise a dicistron encoding an α-galactosidase and a β-glucosidase, respectively, and are predicted to be part of the putative PUL22 involved in arabinan degradation (Supplementary Fig. 7b)^57^. The genes seemingly repressed by GibS (de-repressed in ∆*gibS*) included *BT_1655* (encoding a hypothetical protein within the CPS5 locus; Supplementary Fig. 7c) and *BT_3893* (the second gene within a dicistron and encoding a hypothetical protein; Supplementary Fig. 7e). *BT_3892*—the first gene in this operon and encoding a branched-chain amino acid aminotransferase—was unaffected by *gibS* status. Finally, the glycogen synthesis *BT_0769*–*71* operon, which is encoded adjacent to *gibS* itself (Fig. 4a), was de-repressed in the ∆*gibS* mutant (Supplementary Fig. 7d). We rule out polar effects of sRNA deletion, as *BT_0769*–*71* expression reverted to wild-type levels when GibS was re-introduced in *trans* in the ∆*gibS* background (strain *gibS* + in Fig. 5a). Altered expression levels of all target candidates except *BT_1655* and *BT_0769* were independently validated by quantitative real-time PCR (qRT-PCR) (Fig. 5b).

To test whether the observed GibS-dependent expression changes result from direct sRNA:mRNA base-pairing events, we searched for regions within the target candidates that showed partial complementarity to the GibS sequence. Among the differentially expressed genes identified above, the IntaRNA program^58^ predicted GibS to anneal within the CDS of *BT_1871* and the region spanning the respective start codons of *BT_0771* and *BT_3893* (Fig. 5c). The correspondingly predicted seed region of GibS comprised its highly conserved and unstructured 5′ portion (Fig. 4c). To experimentally test these predictions, we performed electrophoretic mobility shift assays (EMSAs) using radiolabeled GibS and increasing concentrations of ~150 nt-long mRNA segments encompassing the assumed targeting sites. Binding of GibS to the 5′ region of both *BT_3893* and *BT_0771* occurred with high affinity (*K*_d_ = 16.01 and 18.06 nM) resulting in an upshift of the sRNA with increasing concentrations of each mRNA target segment (Fig. 5d; Supplementary Fig. 8a). This suggests *BT_3893* and *BT_0771* mRNAs to be direct targets of GibS. Indeed, in-line probing of radiolabeled GibS with or without in vitro-transcribed *BT_3893* mRNA (Fig. 5e) validated the computationally predicted base-pairing events involving two adjacent regions in the 5′ portion of GibS (termed R1 and R2 in Fig. 5c), and mutating a hexameric sequence within the *BT_3893* region targeted by R2 reduced its affinity to GibS (Fig. 5d; Supplementary Fig. 8b). In contrast, the in vitro interaction of GibS with *BT_1871*, for which binding was proposed to occur within the CDS, was of very low affinity (Supplementary Fig. 8c). This argues that in the bacterial cell, base-pairing between GibS and *BT_1871* may depend on auxiliary factors, such as a RNA chaperone, or that activation of this gene by GibS (Fig. 5a, b) is indirect.

## Discussion

Only recently and thanks to technological breakthroughs that allow anaerobic culturing without the need for extensive lab equipment^59^ and the development of versatile protocols for genetic manipulation^56,60–62^, has it become possible for a wider group of researchers to functionally characterize anaerobic gut commensals. In this context, *Bacteroides* species—predominant members of the human microbiota—are gaining increasing attention by the scientific community^63^.

In the present work, we have compiled a high-resolution transcriptome map of the *B*. *thetaiotaomicron* type strain VPI-5482, which is freely accessible to the research community as an intuitive online database. Our screen identified ~4500 TSSs within the core genome and plasmid, and revealed high plasticity in the transcriptome structure of *B*. *thetaiotaomicron*. Inspection of the sequences upstream of the identified TSSs revealed the canonical σ^ABfr^ promoter. Not only did our genome-wide motif search confirm the –7 box, but it also identified the –33 box that was initially inferred from manual inspection of a few dozen promoters^6^, but could not previously be verified globally within the Bacteroidetes phylum^8^. We assume that the variable spacing between the two boxes (Fig. 1d) might have hampered previous motif searches. In addition, our global promoter analysis revealed a second sequence motif enriched in front of oxygen tension-related genes, suggesting the motif could be recognized by a stationary phase-inducible alternative sigma factor to pre-adapt microbes against exposure to reactive oxygen species^64^. However, this motif differs substantially from the recognition sequence of the sigma factor EcfO, which protects *B*. *fragilis* against oxidative stress^9^ and deserves further investigation.

Comprehensive transcriptomic analyses are reliant on an accurate genome annotation. We intend to regularly update Theta-Base with the most recently reported genomic features. In addition, in the future, plasticity of the *B*. *thetaiotaomicron* transcriptome may be refined even further by similar RNA-seq studies under additional experimental settings, including growth on defined carbon sources or in face of specific stress conditions. For example, given that *Bacteroides* promoter inversions are commonly observed under stressful conditions such as antibiotics exposure or gut adaptation^65^, those data would be useful to explore the effects of invertible *Bacteroides* promoters on global gene expression.

In addition to bacterial transcriptional control networks, post-transcriptional regulation mediated by regulatory RNA molecules has been revealed as a second layer of adapting global gene expression to changing environmental and intrinsic cues. Particularly, *trans*-encoded sRNAs have been described in species across the bacterial phylogenetic tree^15,53^, where they regulate target mRNAs through imperfect base-pairing interactions mediated by short seed sequences and, in many Gram-negative species, dedicated RNA chaperones^66^. In the majority of the described cases, sRNA annealing to the SD sequence and/or start codon, interferes with translation initiation and induces target decay^15^. Conversely, certain sRNAs may promote translation of their target by unfolding inhibitory structures that otherwise block ribosome binding^67,68^. In addition, activating *trans*-encoded sRNAs in γ**-**proteobacteria may induce target gene expression by binding to nascent mRNA leaders and interfering with Rho-mediated premature transcription termination^69,70^. Besides targeting mRNAs, some sRNAs may bind to, and titrate, regulatory RBPs, thereby indirectly controlling the levels of the target mRNA set of the bound protein^71^. Conceptually related, an emerging class of sRNA sponges bind and titrate other sRNAs, creating complex post-transcriptional gene expression control networks^72^. In contrast, if and how *trans*-encoded sRNAs regulate target gene expression in *Bacteroides* has previously barely been addressed.

Overall, our study provides evidence for the existence of >200 noncoding RNAs from diverse classes in *B*. *thetaiotaomicron*. As an example, our screen experimentally validated the 6S RNA homologue of *Bacteroides* and provided evidence for the existence of pRNAs that rescue sequestered RNA polymerases, further highlighting 6S RNA-mediated regulation of transcriptional activity to be an ultra-conserved mechanism. Taking into account that dRNA-seq was performed in just three defined growth stages in a single (rich) medium, the reported numbers for *cis*-antisense (78) and intergenic sRNA candidates (124, of which we consider 49 as high-confidence) are probably conservative. In addition, the screen identified 21 UTR-derived sRNA candidates, suggesting that evolution of dual-function transcripts^73^ is not restricted to Proteobacteria. The full repertoire of *B*. *thetaiotaomicron* noncoding RNAs therefore appears consistent with the numbers reported in other bacteria^20,29,74–77^. However, we want to point out that antisense transcription might at least partially be due to the appearance of random promoters^26^, which is particularly likely in a genome with a low GC content. Consequently, not all identified *cis*-antisense RNA candidates might actually be functional.

Here, we began to mechanistically characterize the GibS sRNA. We selected GibS for several reasons: (i) its strong sequence conservation (especially in its 5′ portion) within the *Bacteroides* genus argues for functionality; (ii) the presence of PM2 upstream of its TSS renders it a representative for a larger set of transcripts; (iii) the generally high steady-state levels of this sRNA—particularly in stationary growth phase and in the presence of the simple sugar GlcNAc—enables robust detection and analysis; and (iv) *gibS* does not overlap with any other gene, which allowed for the straight-forward construction of a clean deletion mutant. In the course of our studies, we validated the TSS of GibS predicted by dRNA-seq and determined the secondary structure of the sRNA. Unlike many well-characterized *trans*-encoded sRNAs in Proteobacteria, GibS contains long single-stranded regions, particularly in its 5′ part. Expression profiling of wild-type *B*. *thetaiotaomicron* and a ∆*gibS* mutant identified potential target mRNAs of this sRNA and provided a possible explanation for the lack of structure at the 5′ end: GibS harbors an extended seed region, comprising the first 30-50 nt of its 5′ portion, that mediates base-pairing with the region around the target mRNA’s start codon (*BT_0771*, *BT_3893*) or, potentially, a region deep within the CDS of *BT_1871*.

What is the physiological role of this sRNA? We find GibS expression to peak when *B*. *thetaiotaomicron* is cultured in the presence of GlcNAc as the sole carbon source. This monosaccharide is a constituent of host-derived glycosaminoglycans such as chondroitin sulfate (CS), dermatan sulfate, and heparin/heparan sulfate (HS), with CS and HS being the priority nutrients for *B*. *thetaiotaomicron*^78,79^. The identified GibS targets are related to metabolic processes: The *BT_1871*-*BT_1872* operon, which is activated by GibS, encodes a galactosidase and a periplasmic glucosidase, and *BT_0770*-*BT_0771*, that is repressed by GibS, encodes a glucan-branching enzyme. Together, this suggests a regulatory network, wherein sensing of a specific glycosaminoglycan-derived monosaccharide results in the induction of GibS, which in turn leads to post-transcriptional metabolic rearrangements in the bacterial cell (Fig. 5f). If true, this places GibS among the growing number of sRNAs involved in carbon catabolite repression in diverse bacterial organisms^80^. Dissecting the exact role of GibS, however, requires further work.

GibS targeting the translation initiation region of *BT_0771* and *BT_3893* mRNAs is reminiscent of the classical mechanism of sRNA-mediated target control, preventing ribosome loading and interfering with translation initiation, often accompanied by enhanced target mRNA decay^15^. We here provided the first example showing that, despite the lack of a SD sequence, Bacteroidetes mRNAs may still be repressed by sRNAs that anneal to their translation initiation region. Consequently, deleting *gibS* from the *B*. *thetaiotaomicron* genome resulted in an upregulation of both these mRNAs, and *trans*-complementation reverted their expression to wild-type levels. In contrast, sRNA targeting within the CDS is relatively rare, but could mask an endonucleolytic cleavage site, thereby stabilizing the target mRNA^68^. In line with such a mechanism, *BT_1871* levels dropped in the absence of GibS as compared to both the wild-type and complementation strain. Alternatively, activation of *BT_1871* mRNA in presence of GibS may occur indirectly.

Commonly, protein co-factors are involved in sRNA-mediated target control^81^. In *Bacteroides*, in absence of an obvious homologue of both Hfq and ProQ, sRNAs might either regulate targets in a protein-independent manner, or depend on an alternative, elusive RNA chaperone. The average GC content of the identified intergenic sRNAs was relatively low (~35%). Moreover, we identified several *B. thetaiotaomicron* sRNAs that appear rather unstructured (Supplementary Fig. 3b). Well-studied Hfq-dependent sRNAs in Proteobacteria typically contain seeds of ~6–10 nt in length^82^. The above features—AT richness and single-strandedness—might indicate that certain *B. thetaiotaomicron* sRNAs need more extended seeds for efficient target annealing, as is exemplified by GibS. Whether such extensive base-pairing events would be more or less likely to require assistance by RNA chaperones needs further investigation. While in the present case, GibS interactions with the 5′ region of *BT_0771* and *BT_3893* occurred at very high affinities even without a protein co-factor in vitro, long single-stranded seed regions may be inherently vulnerable to ribonucleases (homologs of RNase BN, –G, –HII, –III, –P, –R, and –Z are present in *B*. *thetaiotaomicron* according to Pfam^83^) and stability of the respective sRNAs in the bacterial cytosol could thus depend on RBP association.

Compared to species that have long served us as model organisms for prokaryotic RNA biology, there is currently no toolbox available to mechanistically decipher the functions of sRNAs in *Bacteroides* species. Here, we employed an aTC-triggered sRNA induction system, but leaky expression and limited dynamic range (Supplementary Fig. 6) prompted us to grow the respective strain (*gibS*+) for 2 h in presence of the inducer to reach high sRNA levels. Despite successfully complementing expression changes in the ∆*gibS* mutant, more tightly inducible sRNA pulse-expression systems might be harnessed in the future to facilitate discrimination between direct and indirect effects of sRNA overexpression^84,85^. For target validation, two-plasmid systems, consisting of an inducible sRNA expression vector and a constitutively expressed fluorescent reporter fusion with the respective target mRNA, are routinely used in Proteobacteria^86,87^. Despite the oxygen-dependent maturation of commonly used fluorescent proteins, similar reporters should also work in anaerobic bacteria because for sample preparation, cell suspensions are typically shifted to normoxic conditions that allow for fluorescence recovery^88^. Similarly, luciferase assays—albeit depending on molecular oxygen—have already been successfully employed in *Bacteroides*^47,89^ and β-galactosidase assays with *lacZ*-target fusions^90^ represent an oxygen-independent alternative.

Modern sequencing-based technologies have proven useful to gain global perspectives on bacterial sRNAs^91^ and their generic nature should allow sRNA systems biology approaches to be applied to currently understudied bacterial species. For example, pooled knockdown or knockout mutant libraries represent invaluable tools for global screens. Transposon-sequencing (Tn-seq) data exist for *B*. *thetaiotaomicron*^92–94^ and it will be intriguing to re-analyze them for phenotypes associated with a disruption of the here-identified noncoding RNA candidates. However, like any random mutagenesis approach, Tn-seq is inherently biased toward longer genes, typically resulting in an underrepresentation of sRNA mutants. In this context, targeted approaches such as CRISPR interference, whose applicability was already demonstrated for *B*. *thetaiotaomicron*^89^, appear as promising alternatives to simultaneously knock down hundreds of sRNAs and screen the resulting mutant pool under a variety of conditions. Obviously, implementing these technologies requires time and effort. The here-presented data, however, chart a rich world of *Bacteroides* RNA biology for us to explore.

## Methods

### Bacterial strains and genetics

Strains, plasmids, and oligonucleotides used in this study are listed in Supplementary Data 4. *B*. *thetaiotaomicron* type strain VPI-5482 is referred to as wild-type throughout the study. The *gibS* deletion mutant (Δ*gibS*) was generated as previously described^95^. Briefly, 1-kb sequences flanking the region to be deleted were amplified by PCR (using oligos AWO-053/−054, AWO-055/−56, AWO-314/−315, and AWO-316/−317) and assembled into the suicide vector pExchange-*tdk* by Gibson assembly (NEB) as per the manufacturer’s protocol. A 2-μL aliquot of this reaction was transformed into electro-competent *E. coli* S17-1 λpir. Transformants were conjugated with a *tdk* deletion mutant of *B. thetaiotaomicron* (Δ*tdk*) and conjugants counter-selected on 5-fluoro-2′-deoxyuridine (FUdR) plates. Single recombinants were selected on Brain Heart Infusion Supplemented (BHIS) agar containing 200 μg mL^−1^ gentamicin and 25 μg mL^−1^ erythromycin. Double recombinants, resulting in either scarless deletion mutants or wild-type revertants, were selected by growth on BHIS agar containing 200 μg mL^−1^ FUdR and an inability to grow on BHIS agar containing 25 μg mL^−1^ erythromycin. Successful deletions were subsequently confirmed by PCR (AWO-150/−151, AWO-111/−112, AWO-318/−319, AWO-340/−341, AWO-342/−343) and Sanger sequencing.

A *gibS* complementation strain (*gibS*+) was constructed using a variant of the pNBU2 vector system^95^ (Supplementary Data 4). The full *gibS* gene was integrated into the vector by Gibson assembly (AWO-156/−157) to ensure transcription from its native TSS. This resulted in a deletion of the proximal *tetO2* (T_P_) operator downstream of the promoter P1T_DP_, while leaving the second operator (T_D_) intact^56^. This construct, expressing the GibS sRNA from its +1 nucleotide under control of the P1T_D_ promoter, was conjugated into the Δ*tdk* strain and selected on BHIS agar containing 25 μg mL^−1^ erythromycin. Successful insertion was confirmed by PCR (AWO-160/−161) and Sanger sequencing.

### *B*. *thetaiotaomicron* culture conditions

*Bacteroides* strains were cultured in an anaerobic chamber (Coy Laboratory Products) in presence of an anoxic gas mix (85% N_2_, 10% CO_2_, 5% H_2_) at 37 °C. Routine cultivation involved the use of complex media; TYG (20 g L^−1^ tryptone, 10 g L^−1^ yeast extract, 0.5% glucose, 5 mg L^−1^ hemin, 1 g L^−1^ cysteine, 0.0008% CaCl_2_, 19.2 mg L^−1^ MgSO_4_.7H_2_O, 40 mg L^−1^ KH_2_PO_4_, 40 mg L^−1^ K_2_HPO_4_, 80 mg L^−1^ NaCl, 0.2% NaHCO_3_) and BHIS (52 g L^−1^ BHI agar powder, 1 g L^−1^ cysteine, 5 mg L^−1^ hemin, 0.2% NaHCO_3_). Carbohydrate growth assays were performed in minimal medium (1 g L^−1^ L-cysteine, 5 mg L^−1^ hemin, 20 mg L^−1^ L-methionine, 4.17 mg L^−1^ FeSO_4_, 0.2% NaHCO_3,_ 0.9 g L^−1^ KH_2_PO_4_, 0.02 g L^−1^ MgCl_2_.6H_2_O, 0.026 g L^−1^ CaCl_2_.2H_2_O, 0.001 g L^−1^ CoCl_2_.6H_2_O, 0.01 g L^−1^ MnCl_2_.4H_2_O, 0.5 g L^−1^ NH_4_Cl, 0.25 g L^−1^ Na_2_SO_4_) supplemented with 0.5% of the indicated carbon sources^96^.

### RNA extraction, TEX treatment, cDNA library preparation, and sequencing

For dRNA-seq (Figs. 1–3), wild-type *B. thetaiotaomicron* VPI-5482 (AWS-001) was grown overnight in 5 mL pre-reduced TYG medium followed by sub-culturing (1:100) into 50 mL fresh pre-reduced TYG. Total RNA was isolated by hot phenol extraction from culture aliquots of each three biological replicates at early exponential (4 h), mid-exponential (7 h), and stationary growth phase (10 h). Briefly, 4 OD equivalents of culture were harvested and 1.6 mL stop mix^97^ was added (95% vol vol^−1^ ethanol, 5% vol vol^−1^ water saturated phenol, pH >7.0). The bacterial cells were lysed by incubation with 600 μL lysozyme (0.5 mg mL^−1^) and 60 μL 10% SDS for 2 min at 64 °C, followed by the addition of 66 μL of 3 M NaOAc. Phenol extraction (750 μL; Roti-Aqua phenol) was performed at 64 °C for 6 min with the subsequent addition of 750 µL chloroform. RNA was precipitated from the aqueous phase with twice the volume of 30:1 (ethanol:3 M NaOAc, pH 6.5) mix and incubated at –80 °C overnight. After centrifugation, pellets were washed with 75% (vol vol^−1^) ethanol and re-suspended in 50 μL H_2_O. Contaminating genomic DNA was removed by treating 40 μg total RNA with 5 U of DNase I (Fermentas) and 0.5 μL Superase-In RNase Inhibitor (Ambion) in a 50 μL reaction. RNA quality was checked using a 2100 Bioanalyzer and the RNA 6000 Nano kit (Agilent Technologies). RNA integrity (RIN) values for all samples were between 9.2 and 9.6.

Prior to cDNA synthesis, total RNA was fragmented using ultrasound (4 pulses of 30 s at 4 °C) and treated with T4 polynucleotide kinase (NEB). Subsequently, half of each total RNA sample was treated with Terminator exonuclease (TEX) to enrich for primary transcripts, whereas the other half was left untreated. RNA samples were then poly(A)-tailed using poly(A) polymerase and 5′-PPP was removed with 5′ polyphosphatase (Epicentre). RNA adaptors were ligated and first-strand cDNA synthesis was carried out using oligo(dT) primers and M-MLV reverse transcriptase. The cDNA was PCR amplified to about 10-20 ng μL^−1^, purified using Agencourt AMPure XP kit (Beckman Coulter Genomics), and fractionated in a size range of 200–500 bp. Libraries were sequenced on an Illumina NextSeq platform (150 cycles) at the Core Unit SysMed of the University of Würzburg.

For conventional RNA-seq (Fig. 5a), *B*. *thetaiotaomicron* ∆*tdk* (AWS-003), ∆*gibS*, (AWS-028), and *gibS*+ (AWS-035) strains (two biological replicates) were grown in TYG to stationary phase as described above, followed by the addition of aTC at a final concentration of 200 ng mL^−1^ (for maximal GibS expression with minimal growth attenuation; Supplementary Fig. 6a, b), and resumed growth therein for 2 h. Total RNA was extracted and RNA quality checked as above with RIN values between 7.7 and 9.5. Prior to library preparation, rRNA was depleted using the Pan-Prokaryote riboPOOLs kit (siTOOLs Biotech). In brief, 1 µg of total RNA was incubated for 10 min at 68 °C and 30 min at 37 °C with 100 pmol of rRNA-specific biotinylated DNA probes in 2.5 mM Tris-HCl pH 7.5, 0.25 mM EDTA, and 500 mM NaCl. DNA-rRNA hybrids were depleted from total RNA by two consecutive 15 min incubations with 0.45 mg streptavidin-coated magnetic Dynabeads MyOne C1 (ThermoFisher Scientific) in 2.5 mM Tris-HCl pH 7.5, 0.25 mM EDTA, and 1 M NaCl at 37 °C. The rRNA-depleted RNA samples were purified using the Zymo RNA Clean & Concentrator kit combined with DNase treatment on a solid support (Zymo Research).

cDNA libraries were prepared using the NEBNext Multiplex Small RNA Library Prep kit for Illumina (NEB) in accordance with the manufacturers’ instructions, except for the following modifications: RNA samples were fragmented with Mg^2+^ for 2.75 min at 94 °C using the NEBNext Magnesium RNA Fragmentation Module (NEB) followed by RNA purification with the Zymo RNA Clean & Concentrator kit. Fragmented RNA was dephosphorylated at the 3′ end, phosphorylated at the 5′ end, and decapped using 10 U T4-PNK +/− 40 nmol ATP and 5 U RppH, respectively (NEB). After each enzymatic treatment, RNA was purified with the Zymo RNA Clean & Concentrator kit. RNA fragments were ligated for cDNA synthesis to the 3′ SR and 5′ SR adapters pre-diluted 1:3 with nuclease-free water. PCR amplification to add Illumina adaptors and indices to the cDNA was performed for 14 cycles with 1:3 pre-diluted primers. Barcoded cDNA libraries were purified using magnetic MagSi-NGSPREP Plus beads (amsbio) at a 1.8:1 ratio of beads to sample volume. Libraries were quantified with the Qubit 3.0 Fluometer (ThermoFisher) and the library quality and size distribution was checked using a 2100 Bioanalyzer with the high sensitivity DNA kit (Agilent). Sequencing of ten pooled libraries spiked with 5% PhiX control library was performed in single-end mode on the NextSeq 500 platform (Illumina) with the Mid Output Kit v2.5 (75 cycles).

### Read processing and mapping

Sequencing reads were quality filtered with the local run manager software from Illumina version 2.2.0. Generated reads were then trimmed for the NEBNext adapter sequence using Cutadapt version 2.5 with default parameters. In addition, Cutadapt was given the –nextseq-trim=20 switch to handle two-color sequencing chemistry and reads that were trimmed to length 0 were discarded.

For both sequencing protocols (dRNA-seq and conventional RNA-seq), reads were mapped to the *B*. *thetaiotaomicron* VPI-5482 reference genome (NC_004663.1) and plasmid (NC_004703.1) using the READemption pipeline^98^. Details of alignment statistics can be found in Supplementary Data 5. Aligned reads in wiggle format were visualized using both the Integrated Genome Browser^99^ and JBrowse^43^. Differential gene expression analysis was performed using DESeq2^100^ with log fold-change shrinkage using the DESeq2 betaprior method.

### Identification of TSSs

We employed the ANNOgesic pipeline (version 0.7.33) that integrates a suite of tools to annotate bacterial genomes from both dRNA-seq and conventional RNA-seq data^22^. TSSs were identified using the ANNOgesic implementation of TSSpredator^77^ (usage: annogesic tss_ps) which compares the relative enrichment of reads between TEX-treated samples and their untreated counterparts. This resulted in characteristic enrichment peaks that were indicative of a 5′ triphosphate that protects against TEX digestion. Default settings were used with the addition of the gene validation option that relates identified TSSs to annotated genes (Supplementary Data 1). TSSs were categorized based on their enrichment and location relative to the start of the cognate coding gene. Primary TSSs were classified as having the highest coverage within 300 bp upstream of an ORF, while all other TSSs within this region were defined as secondary TSSs. Internal TSSs were identified as originating on the sense strand within a coding gene and antisense TSSs were located on the antisense strand overlapping with, or within a 100 bp flanking region, of a given sense gene. All remaining TSSs were classified as orphan TSSs. TSSs called by TSSpredator were manually curated based on read coverage plots to ensure accuracy of the assignments and the thus validated TSSs are incorporated in Theta-Base (www.helmholtz-hiri.de/en/datasets/bacteroides).

### Search for promoter motifs

Conserved DNA sequences upstream of TSSs were identified by ANNOgesic (usage: annogesic promoter) integrating both, MEME for gapless motifs^38^ and GLAM2 for gapped motifs^39^. Default parameters were used with the addition of the flag –n set to 50 to increase sensitivity. Representative sequences containing consensus motifs were aligned using ClustalW (version 2.1)^101^ and logos generated with WebLogo (version 2.8)^102^.

### Prediction of intrinsic terminators

ANNOgesic employs two heuristic algorithms for the prediction of Rho-independent terminators (usage: annogesic terminator). TransTermHP scans genome sequences for the presence of Rho-independent terminators^30^. To further substantiate these predictions and to detect the presence of Rho-independent terminators also in between convergent gene pairs, RNA-seq data were referenced to detect a significant decrease in coverage associated with the predicted terminators. All parameters were run at default values. The combined results of these predictions are available in Supplementary Data 6 and incorporated into Theta-Base (www.helmholtz-hiri.de/en/datasets/bacteroides).

### sRNA identification

To detect putative sRNAs in *B*. *thetaiotaomicron* from our dataset, ANNOgesic (usage: srna) tested transcripts for several criteria. First, detected transcripts were compared with RNAs contained within the sRNA database (BSRD; http://www.bac-srna.org/BSRD/index.jsp) and the non-redundant protein database (nr database; ftp://ftp.ncbi.nih.gov/blast/db/FASTA/). Homologs identified in the former were classified as putative sRNAs, while those contained in the latter were excluded from further analysis.

The remaining transcripts were further assessed and classified as intergenic sRNA candidates in case of the presence of a defined TSS, a stable secondary structure (folding energy change as calculated by RNAfold and normalized to sRNA length < –0.05), and a length ranging from 30 to 500 nt. *Cis*-antisense RNAs were identified in a similar manner except that in addition to the above criteria, they originated in antisense orientation to annotated genes. sRNAs sharing a TSS with a mRNA were classified as 5′ UTR-derived if they were associated with a sharp drop in coverage and/or a processing site in front of the cognate CDS. 3′ UTR-derived sRNAs required a TSS or processing site within the 3′ region of the cognate coding gene and either a processing site or shared terminator with the parental mRNA. Intra-operonic sRNAs required a TSS or processing site at the 5′ end as well as a coverage drop or processing site at their 3′ end.

To maximize detection of sRNAs while minimizing the number of false positives, we used a list of known or proposed *cis*-antisense RNAs from a previous study^18^ as a benchmark and lowered the minimum average coverage to 5. With this modification, ANNOgesic recovered five out of seven benchmark RNAs (Supplementary Data 2). The high success rate of experimental validation for the identified sRNA candidates from all categories (Fig. 3d) further supports the predictions.

### sRNA conservation analysis

We constructed a custom genome database consisting of completed bacterial genomes from the ENA (https://www.ebi.ac.uk/genomes/bacteria.html, accessed 1/12/2017) belonging to class Bacteroidia according to the NCBI Taxonomy (Taxonomy ID: 200643). We then performed an iterative search for each candidate sRNA sequence using nhmmer 3.1b1^103^, similarly to as previously described^104^. In each round of iteration nhmmer was run with the flags --popen 0.4999 -*E* 0.001 --inc*E* 0.001, hit sequences with an *E* value of 0.001 or less were additionally required to have near-full length alignments not missing more than 10% of sequence length at either end, and the resulting alignment was used as input for hmmbuild and fed into the next round of iteration. The alignments were then manually examined using the RALEE alignment editor^105^. The final homologous sequences identified for GibS were additionally subjected to realignment and structure prediction using the webserver for aligning structural RNAs (WAR) to improve prediction of secondary structure^52^.

### Northern blotting and qRT-PCR analysis

The sequences of DNA oligonucleotides used for Northern blot and qRT-PCR are given in Supplementary Data 4. Northern blotting was performed as described previously^106^. Briefly, total RNA (2.5–10 μg) was run on a 6% (vol vol^−1^) polyacrylamide (PAA)-7 M urea gel and electro-blotted onto Hybond XL membranes (Amersham) at 50 V, 4 °C for 1 h. Blots were probed with ^32^P-labeled gene-specific oligonucleotides in Hybri-Quick buffer (Carl Roth AG) at 42 °C and exposed as required. Visualization was achieved using a phosphorimager (FLA‐3000 Series, Fuji) and images were quantified using ImageJ^107^.

For qRT-PCR assays, reverse transcription and PCR amplification were performed in the same reaction mix containing 1 µL of DNase I-treated RNA (adjusted in water to 10 ng µL^−1^), 5 µL master mix (No ROX SYBR MasterMix blue dTTP kit, Takyon), 0.1 µL of forward and reverse primer (10 µM each), and 0.08 µL reverse transcriptase (One-Step Kit converter, Takyon) per well in a 96-well format. Two technical replicates per each biological replicate were pipetted and plates analyzed on a CFX96 instrument (Biorad).

### Primer-extension analysis

Primer-extensions were performed in 20 µL reactions containing 9 µL (10 µg) of DNase I-treated total RNA and 1 µL of 5′ end-labeled DNA oligonucleotide (AWO-348). After an initial denaturation step at 95 °C for 1 min followed by a 5-min incubation on ice, 10 µL of the elongation mix (4 µL 5x first-strand buffer, 0.5 mM dNTP mix, 0.5 µL RNase Inhibitor, and 5 mM DTT) were added to the sample and incubated for 5 min at 42 °C for annealing to occur. Thereafter, 1 µL of the reverse transcriptase (1:1 dilution; SuperScript III, Thermo Fischer Scientific) was added, followed by an incubation for 1 h at 50 °C. The reaction was terminated at 70 °C for 15 min, followed by the addition of 1 µL RNase H. For preparation of the sequencing ladder, oligonucleotides (AWO-347/−348) were used to PCR-amplify the region –50 nt to +87 nt relative to the TSS of GibS from genomic DNA. A sequencing reaction was set up using the labeled oligonucleotide and the DNA cycle sequencing kit (Jena Bioscience) according to the manufacturers’ instructions. Both, sample and sequencing ladder (10 µL each) were electrophoresed on a sequencing gel (10% [vol vol^−1^] PAA-7 M urea). The gel was dried, exposed, and visualized on a phosphorimager (FLA‐3000 Series, Fuji).

### In vitro transcription and radiolabeling of RNA

Primer pairs carrying a T7 promoter and amplifying templates from genomic DNA are listed in Supplementary Data (AWO-241/−311, AWO-329/−313, AWO-331/−332, AWO-333/−334). Similarly, a hexanucleotide substitution (UAAUCU → AUUAGA) in the R2-targeted segment of *BT_3893* (‘mut’) was generated by overlap extension PCR using primer pairs AWO-329/−369 and 359/−313. In vitro transcription was carried out using the MEGAscript T7 kit (Ambion) followed by DNase I digestion (1 U, 37 °C for 15 min). The in vitro-transcribed RNA product was extracted from a 6% (vol vol^−1^) PAA-7M urea gel by comparison to the LowRange RNA ladder (ThermoFisher Scientific) and subsequently eluted in RNA elution buffer (0.1 M NaOAc, 0.1% SDS, 10 mM EDTA) overnight in a thermoblock at 8 °C with shaking at 1400 rpm. The RNA was precipitated using ethanol:NaOAc (30:1) mix, washed with 75% ethanol, and re-suspended in 20 µL water (65 °C for 5 min).

For radioactive labeling, 50 pmol of the in vitro transcript were dephosphorylated using 25 U of calf intestine alkaline phosphatase (NEB) in a 50 µL reaction volume and incubated for 1 h at 37 °C. Following extraction with phenol:cholorform:isoamylalcohol (P:C:I, 25:24:1), the RNA was precipitated as described above. The dephosphorylated RNA (20 pmol) was 5′ end-labeled (20 µCi of ^32^P-γATP) using 1 U of polynucleotide kinase (NEB) for 1 h at 37 °C in a 20 µL reaction volume. The labeled RNA was purified on a G-50 column (GE Healthcare) and extracted from a PAA gel as above.

### Structure and in-line probing

Structure probing was carried out in 10 μL reactions^108^. Briefly, 5′ end-labeled RNA (0.2 pmol) was denatured at 95 °C for 1 min and chilled on ice for 5 min. 1 μg of yeast RNA was added followed by the addition of 10x structure buffer (Ambion) and the reaction incubated at 37 °C for 10 min, prior to the addition of 2 μL of freshly prepared lead (II) acetate (25 mM; Fluka), 2 μL of RNase T1 (0.01 U μL^−1^; Ambion) or 2 μL of RNase III (New England Biolabs) and 1 mM DTT. The reactions were incubated for 45 s (lead [II] acetate), 3 min (RNase T1) or 10 min (RNase III), respectively, at 37 °C, stopped by adding 12 μL gel loading buffer II (Ambion), and stored on ice. The alkaline hydrolysis ladder was prepared by incubating 0.4 pmol labeled RNA with 9 μL 1x alkaline hydrolysis buffer (Ambion) and incubated at 95 °C for 5 min. The RNase T1 ladder was prepared by incubating 0.4 pmol labeled RNA in 8 μL of 1x sequencing buffer (Ambion) at 95 °C for 1 min followed by the addition of 1 μL RNase T1 (0.1 U µL^−1^) and incubation at 37 °C for 5 min. Both reactions were stopped by the addition of 12 μL loading buffer II and stored on ice. Immediately prior to loading, the samples were denatured at 95 °C for 3 min and resolved on an 8% (vol vol^−1^) PAA-7 M urea sequencing gel. The gel was visualized after appropriate exposure as described above.

In-line probing assays were performed by incubating 0.2 pmol labeled RNA for 40 h at room temperature in 1x in-line probing buffer (100 mM KCl, 20 mM MgCl_2_, 50 mM Tris-HCl, pH 8.3). RNase T1 and alkaline hydrolysis ladders were prepared as mentioned above. Reactions were stopped by the addition of 10 µL colorless loading dye (1.5 mM EDTA, pH 8, 10 M urea) on ice. Samples were run on a 10% (vol vol^−1^) PAA-7 M urea sequencing gel and visualized as described above.

### Electrophoretic mobility shift assays

EMSAs were performed in 10 μL reactions as described^109^, containing 1x RNA structure buffer (SB; Ambion), 5′ end-labeled GibS RNA (4 nM final concentration), 1 μg yeast RNA (~4 μM final concentration), and putative target mRNA segments (~150 nt in length) at the following final concentrations: 0, 8, 16, 32, 64, 128, 256, 512, and 1024 nM. The reactions were incubated at 37 °C for 1 h following which 3 μL of 5x native loading dye (0.2% bromophenol blue, 0.5x TBE, 50% glycerol) were added. Electrophoresis of the samples was carried out on a native 6% (vol vol^−1^) PAA gel in 0.5x TBE buffer at 4 °C and 300 V, for 3 h. The gels were dried and visualized as above.

### Theta-Base

The Theta-Base website provides an interactive tool to interrogate the transcriptome structure and gene expression profile of *B*. *thetaiotaomicron* type strain VPI-5482 as determined in the course of this work. The user interface is implemented in Python using Dash by Plotly^42^. The Dash app uses the Flask web framework for the back end and is deployed with Gunicorn, a Python WSGI HTTP Server for UNIX. The experimental data are stored in an efficient SQLite database^110^. The app offers the possibility to create heatmaps of user- or pre-defined lists of coding or noncoding RNAs over the experimental conditions tested in this work (early exponential, mid-exponential, and stationary phase in TYG). The data can be exported to, and modified in, the interactive graphing library Plotly and the heatmaps can be saved. In addition, the website runs an instance of JBrowse^43^ to explore operon structures, the position of coding and noncoding loci, TSSs, and terminators in the *B*. *thetaiotaomicron* transcriptome.

### Reporting summary

Further information on research design is available in the Nature Research Reporting Summary linked to this article.

## Supplementary information

Supplementary Information

Description of Additional Supplementary Files

Supplementary Data 1

Supplementary Data 2

Supplementary Data 3

Supplementary Data 4

Supplementary Data 5

Supplementary Data 6

Reporting Summary

## Data Availability

All sequencing data are available at NCBI Gene Expression Omnibus (http://www.ncbi.nlm.nih.gov/geo) under the accession number GSE144492. The source data underlying Figs. 1a–d, 3c, d, 4d, e, 5a, b, d, e and Supplementary Figs. 1b, d, e, 5, 6a, b, 7a and 8a–c are provided as a Source Data file. Sequences in FASTA format were downloaded from the sRNA database (BSRD; http://www.bac-srna.org/BSRD/index.jsp) and non-redundant protein database (nr database; ftp://ftp.ncbi.nih.gov/blast/db/FASTA/). Complete genome sequences were downloaded from ENA (https://www.ebi.ac.uk/genomes/bacteria.html, accessed 1/12/2017).

## Source data

Source Data

## Code availability

Core software central to the conclusions drawn in this study are publicly available and their usage parameters described in the appropriate sections above.

## References

[1] Wexler HM (2007). *Bacteroides*: the good, the bad, and the nitty-gritty. Clin. Microbiol Rev..

[2] Martens EC (2011). Recognition and degradation of plant cell wall polysaccharides by two human gut symbionts. PLoS Biol..

[3] Lee SM (2013). Bacterial colonization factors control specificity and stability of the gut microbiota. Nature.

[4] Martens EC, Roth R, Heuser JE, Gordon JI (2009). Coordinate regulation of glycan degradation and polysaccharide capsule biosynthesis by a prominent human gut symbiont. J. Biol. Chem..

[5] Martens EC, Chiang HC, Gordon JI (2008). Mucosal glycan foraging enhances fitness and transmission of a saccharolytic human gut bacterial symbiont. Cell Host Microbe.

[6] Bayley DP, Rocha ER, Smith CJ (2000). Analysis of cepA and other *Bacteroides* fragilis genes reveals a unique promoter structure. FEMS Microbiol. Lett..

[7] Vingadassalom D (2005). An unusual primary sigma factor in the Bacteroidetes phylum. Mol. Microbiol..

[8] Baez WD (2019). Global analysis of protein synthesis in *Flavobacterium johnsoniae* reveals the use of Kozak-like sequences in diverse bacteria. Nucleic Acids Res.

[9] Ndamukong IC, Gee J, Smith CJ (2013). The extracytoplasmic function sigma factor EcfO protects *Bacteroides fragilis* against oxidative stress. J. Bacteriol..

[10] D’Elia JN, Salyers AA (1996). Effect of regulatory protein levels on utilization of starch by *Bacteroides thetaiotaomicron*. J. Bacteriol..

[11] Sonnenburg ED (2006). A hybrid two-component system protein of a prominent human gut symbiont couples glycan sensing in vivo to carbohydrate metabolism. Proc. Natl Acad. Sci. USA.

[12] Sonnenburg ED (2010). Specificity of polysaccharide use in intestinal bacteroides species determines diet-induced microbiota alterations. Cell.

[13] Ravcheev, D. A., Godzik, A., Osterman, A. L. & Rodionov, D. A. Polysaccharides utilization in human gut bacterium *Bacteroides thetaiotaomicron*: comparative genomics reconstruction of metabolic and regulatory networks. *Bmc Genomics*10.1186/1471-2164-14-873 (2013). 10.1186/1471-2164-14-873 PMC3878776 24330590

[14] Chang C (2015). A novel transcriptional regulator of L-arabinose utilization in human gut bacteria. Nucleic Acids Res..

[15] Wagner EG, Romby P (2015). Small RNAs in bacteria and archaea: who they are, what they do, and how they do it. Adv. Genet.

[16] Jeters RT, Wang GR, Moon K, Shoemaker NB, Salyers AA (2009). Tetracycline-associated transcriptional regulation of transfer genes of the *Bacteroides* conjugative transposon CTnDOT. J. Bacteriol..

[17] Waters JL, Salyers AA (2012). The small RNA RteR inhibits transfer of the *Bacteroides* conjugative transposon CTnDOT. J. Bacteriol..

[18] Cao Y, Forstner KU, Vogel J, Smith CJ (2016). cis-Encoded small RNAs, a conserved mechanism for repression of polysaccharide utilization in *Bacteroides*. J. Bacteriol..

[19] Townsend GE (2019). Dietary sugar silences a colonization factor in a mammalian gut symbiont. Proc. Natl Acad. Sci. USA.

[20] Sharma, C. M. et al. The primary transcriptome of the major human pathogen *Helicobacter pylori*. *Nature* **464**, 250–255 (2010). 10.1038/nature08756 20164839

[21] Sharma CM, Vogel J (2014). Differential RNA-seq: the approach behind and the biological insight gained. Curr. Opin. Microbiol.

[22] Yu, S. H., Vogel, J. & Forstner, K. U. ANNOgesic: a Swiss army knife for the RNA-seq based annotation of bacterial/archaeal genomes. *GigaScience*10.1093/gigascience/giy096 (2018). 10.1093/gigascience/giy096 PMC6123526 30169674

[23] Mi HY, Muruganujan A, Casagrande JT, Thomas PD (2013). Large-scale gene function analysis with the PANTHER classification system. Nat. Protoc..

[24] Georg, J. & Hess, W. R. Widespread antisense transcription in prokaryotes. *Microbiol. Spectrum*10.1128/microbiolspec.RWR-0029-2018 (2018). 10.1128/microbiolspec.rwr-0029-2018 PMC11633618 30003872

[25] Wade JT, Grainger DC (2014). Pervasive transcription: illuminating the dark matter of bacterial transcriptomes. Nat. Rev. Microbiol.

[26] Llorens-Rico, V. et al. Bacterial antisense RNAs are mainly the product of transcriptional noise. *Sci. Adv.* 10.1126/sciadv.1501363 (2016). 10.1126/sciadv.1501363 PMC4783119 26973873

[27] Ramachandran, V. K., Shearer, N. & Thompson, A. The primary transcriptome of *Salmonella enterica* Serovar Typhimurium and its dependence on ppGpp during late stationary phase. *PLoS ONE*10.1371/journal.pone.0092690 (2014). 10.1371/journal.pone.0092690 PMC3963941 24664308

[28] Berger P (2016). The primary transcriptome of the *Escherichia coli* O104:H4 pAA plasmid and novel insights into its virulence gene expression and regulation. Sci. Rep.-Uk.

[29] Kroger C (2018). The primary transcriptome, small RNAs and regulation of antimicrobial resistance in *Acinetobacter baumannii* ATCC 17978. Nucleic Acids Res..

[30] Kingsford, C. L., Ayanbule, K. & Salzberg, S. L. Rapid, accurate, computational discovery of Rho-independent transcription terminators illuminates their relationship to DNA uptake. *Genome Biol.*10.1186/Gb-2007-8-2-R22 (2007). 10.1186/gb-2007-8-2-r22 PMC1852404 17313685

[31] Lorenz R (2011). ViennaRNA Package 2.0. Algorithms Mol. Biol..

[32] Kim, D. et al. Comparative analysis of regulatory elements between *Escherichia coli* and *Klebsiella pneumoniae* by genome-wide transcription start site profiling. *PLoS Genet.* **8**, e1002867 (2012). 10.1371/journal.pgen.1002867 PMC3415461 22912590

[33] Brock JE, Pourshahian S, Giliberti J, Limbach PA, Janssen GR (2008). Ribosomes bind leaderless mRNA in *Escherichia coli* through recognition of their 5 ‘-terminal AUG. Rna-a Publ. Rna Soc..

[34] Yanofsky C, Konan KV, Sarsero JP (1996). Some novel transcription attenuation mechanisms used by bacteria. Biochimie.

[35] Duval M, Cossart P (2017). Small bacterial and phagic proteins: an updated view on a rapidly moving field. Curr. Opin. Microbiol..

[36] Storz G, Wolf YI, Ramamurthi KS (2014). Small proteins can no longer be ignored. Annu. Rev. Biochem..

[37] Sberro H (2019). Large-scale analyses of human microbiomes reveal thousands of small, novel genes. Cell.

[38] Bailey TL (2009). MEME SUITE: tools for motif discovery and searching. Nucleic Acids Res.

[39] Frith MC, Saunders NF, Kobe B, Bailey TL (2008). Discovering sequence motifs with arbitrary insertions and deletions. PLoS Comput Biol..

[40] Colgan AM, Cameron AD, Kroger C (2017). If it transcribes, we can sequence it: mining the complexities of host-pathogen-environment interactions using RNA-seq. Curr. Opin. Microbiol.

[41] Kroger, C. et al. An infection-relevant transcriptomic compendium for *Salmonella enterica* Serovar Typhimurium. *Cell Host Microbe* **14**, 683–695 (2013). 10.1016/j.chom.2013.11.010 24331466

[42] Inc., P. T. *Collaborative Data Science*, https://plot.ly (2015).

[43] Buels R (2016). JBrowse: a dynamic web platform for genome visualization and analysis. Genome Biol..

[44] Wehner S, Damm K, Hartmann RK, Marz M (2014). Dissemination of 6S RNA among Bacteria. RNA Biol..

[45] Kalvari I (2018). Rfam 13.0: shifting to a genome-centric resource for non-coding RNA families. Nucleic Acids Res.

[46] Costliow, Z. A. & Degnan, P. H. Thiamine acquisition strategies impact metabolism and competition in the gut microbe *Bacteroides thetaiotaomicron*. *Msystems* **2**, e00116–e00117 (2017). 10.1128/mSystems.00116-17 PMC5613172 28951891

[47] Costliow, Z. A., Degnan, P. H. & Vanderpool, C. K. Thiamine pyrophosphate riboswitches in *Bacteroides* species regulate transcription or translation of thiamine transport and biosynthesis genes. Preprint at https://www.biorxiv.org/content/10.1101/867226v1(2019).

[48] Hershberg R, Altuvia S, Margalit H (2003). A survey of small RNA-encoding genes in *Escherichia coli*. Nucleic Acids Res..

[49] Tajkarimi M, Wexler HM (2017). CRISPR-Cas systems in *Bacteroides fragilis*, an important pathobiont in the human gut microbiome. Front. Microbiol..

[50] Bland C (2007). CRISPR recognition tool (CRT): a tool for automatic detection of clustered regularly interspaced palindromic repeats. BMC Bioinforma..

[51] Gottesman S, Storz G (2011). Bacterial small RNA regulators: versatile roles and rapidly evolving variations. Cold Spring Harb. Perspect. Biol..

[52] Torarinsson E, Lindgreen S (2008). WAR: Webserver for aligning structural RNAs. Nucleic Acids Res.

[53] Storz G, Vogel J, Wassarman KM (2011). Regulation by small RNAs in bacteria: expanding frontiers. Mol. Cell.

[54] Schwalm, N. D. III, Townsend, G. E. II & Groisman, E. A. Multiple signals govern utilization of a polysaccharide in the gut bacterium *Bacteroides thetaiotaomicron*. *MBio*10.1128/mBio.01342-16 (2016). 10.1128/mBio.01342-16 PMC5061871 27729509

[55] Bobrovskyy M, Vanderpool CK (2013). Regulation of bacterial metabolism by small RNAs using diverse mechanisms. Annu. Rev. Genet..

[56] Lim B, Zimmermann M, Barry NA, Goodman AL (2017). Engineered regulatory systems modulate gene expression of human commensals in the gut. Cell.

[57] Terrapon N (2018). PULDB: the expanded database of Polysaccharide Utilization Loci. Nucleic Acids Res..

[58] Mann M, Wright PR, Backofen R (2017). IntaRNA 2.0: enhanced and customizable prediction of RNA-RNA interactions. Nucleic Acids Res.

[59] Lagier JC (2018). Culturing the human microbiota and culturomics. Nat. Rev. Microbiol.

[60] Whitaker WR, Shepherd ES, Sonnenburg JL (2017). Tunable expression tools enable single-cell strain distinction in the gut microbiome. Cell.

[61] Garcia-Bayona, L. & Comstock, L. E. Streamlined genetic manipulation of diverse *Bacteroides* and *Parabacteroides* isolates from the human gut microbiota. *MBio*10.1128/mBio.01762-19 (2019). 10.1128/mBio.01762-19 PMC6692515 31409684

[62] Bencivenga-Barry, N. A., Lim, B., Herrera, C. M., Trent, M. S. & Goodman, A. L. Genetic manipulation of wild human gut *Bacteroides*. *J Bacteriol.*10.1128/JB.00544-19 (2019). 10.1128/JB.00544-19 PMC6964735 31712278

[63] Wexler AG, Goodman AL (2017). An insider’s perspective: *Bacteroides* as a window into the microbiome. Nat. Microbiol..

[64] Mishra S, Imlay JA (2013). An anaerobic bacterium, *Bacteroides thetaiotaomicron*, uses a consortium of enzymes to scavenge hydrogen peroxide. Mol. Microbiol.

[65] Jiang X (2019). Invertible promoters mediate bacterial phase variation, antibiotic resistance, and host adaptation in the gut. Science.

[66] Holmqvist, E. & Vogel, J. RNA-binding proteins in bacteria. *Nat. Rev. Microbiol*. **16**, 601–615 (2018). 10.1038/s41579-018-0049-5 29995832

[67] Frohlich, K. S. & Vogel, J. Activation of gene expression by small RNA. *Curr. Opin. Microbiol* **12**, 674–682 (2009). 10.1016/j.mib.2009.09.009 19880344

[68] Papenfort K, Vanderpool CK (2015). Target activation by regulatory RNAs in bacteria. FEMS Microbiol. Rev..

[69] Sedlyarova N (2016). sRNA-mediated control of transcription termination in *E. coli*. Cell.

[70] Silva IJ (2019). SraL sRNA interaction regulates the terminator by preventing premature transcription termination of rho mRNA. Proc. Natl Acad. Sci. USA.

[71] Romeo, T. & Babitzke, P. Global regulation by CsrA and its RNA antagonists. *Microbiol. Spectrum*10.1128/microbiolspec.RWR-0009-2017 (2018). 10.1128/microbiolspec.rwr-0009-2017 PMC5868435 29573256

[72] Bossi L, Figueroa-Bossi N (2016). Competing endogenous RNAs: a target-centric view of small RNA regulation in bacteria. Nat. Rev. Microbiol.

[73] Miyakoshi M, Chao Y, Vogel J (2015). Regulatory small RNAs from the 3′ regions of bacterial mRNAs. Curr. Opin. Microbiol.

[74] Albrecht, M. et al. The transcriptional landscape of *Chlamydia pneumoniae*. *Genome Biol*. **12**, R98 (2011). 10.1186/gb-2011-12-10-r98 PMC3333780 21989159

[75] Toledo-Arana A (2009). The *Listeria* transcriptional landscape from saprophytism to virulence. Nature.

[76] Vogel J (2003). RNomics in *Escherichia coli* detects new sRNA species and indicates parallel transcriptional output in bacteria. Nucleic Acids Res..

[77] Dugar G (2013). High-resolution transcriptome maps reveal strain-specific regulatory features of multiple *Campylobacter jejuni* isolates. PLoS Genet.

[78] Rogers TE (2013). Dynamic responses of *Bacteroides thetaiotaomicron* during growth on glycan mixtures. Mol. Microbiol.

[79] Pudlo NA (2015). Symbiotic human gut bacteria with variable metabolic priorities for host mucosal glycans. mBio.

[80] Durica-Mitic, S., Gopel, Y. & Gorke, B. Carbohydrate utilization in bacteria: making the most out of sugars with the help of small regulatory RNAs. *Microbiol. Spectrum*10.1128/Microbiolspec.Rwr-0013-2017 (2018). 10.1128/microbiolspec.rwr-0013-2017 PMC11633585 29573258

[81] Woodson, S. A., Panja, S. & Santiago-Frangos, A. Proteins that chaperone RNA regulation. *Microbiol. Spectrum*10.1128/microbiolspec.RWR-0026-2018 (2018). 10.1128/microbiolspec.rwr-0026-2018 PMC6086601 30051798

[82] Gorski SA, Vogel J, Doudna JA (2017). RNA-based recognition and targeting: sowing the seeds of specificity. Nat. Rev. Mol. Cell Biol..

[83] El-Gebali S (2019). The Pfam protein families database in 2019. Nucleic Acids Res..

[84] Masse E, Vanderpool CK, Gottesman S (2005). Effect of RyhB small RNA on global iron use in *Escherichia coli*. J. Bacteriol..

[85] Papenfort K (2006). SigmaE-dependent small RNAs of *Salmonella* respond to membrane stress by accelerating global omp mRNA decay. Mol. Microbiol.

[86] Urban JH, Vogel J (2007). Translational control and target recognition by *Escherichia coli* small RNAs in vivo. Nucleic Acids Res..

[87] Corcoran CP (2012). Superfolder GFP reporters validate diverse new mRNA targets of the classic porin regulator, MicF RNA. Mol. Microbiol.

[88] Pinilla-Redondo, R., Riber, L. & Sorensen, S. J. Fluorescence recovery allows the implementation of a fluorescence reporter gene platform applicable for the detection and quantification of horizontal gene transfer in anoxic environments. *Appl. Environ. Microb*. **84**, e02507–e02517 (2018). 10.1128/AEM.02507-17 PMC5835726 29330182

[89] Mimee M, Tucker AC, Voigt CA, Lu TK (2015). Programming a human commensal bacterium, *Bacteroides thetaiotaomicron*, to sense and respond to stimuli in the murine gut microbiota. Cell Syst..

[90] Huntzinger E (2005). Staphylococcus aureus RNAIII and the endoribonuclease III coordinately regulate spa gene expression. EMBO J..

[91] Barquist L, Vogel J (2015). Accelerating discovery and functional analysis of small RNAs with new technologies. Annu. Rev. Genet..

[92] Goodman AL (2009). Identifying genetic determinants needed to establish a human gut symbiont in its habitat. Cell Host Microbe.

[93] Wu M (2015). Genetic determinants of in vivo fitness and diet responsiveness in multiple human gut *Bacteroides*. Science.

[94] Liu, H. P. M. et al. Large-scale chemical-genetics of the human gut bacterium *Bacteroides thetaiotaomicron*. Preprint at https://www.biorxiv.org/content/10.1101/573055v1 (2019).

[95] Koropatkin NM, Martens EC, Gordon JI, Smith TJ (2008). Starch catabolism by a prominent human gut symbiont is directed by the recognition of amylose helices. Structure.

[96] Bacic MK, Smith CJ (2008). Laboratory maintenance and cultivation of *Bacteroides* species. Curr. Protoc. Microbiol..

[97] Eriksson S, Lucchini S, Thompson A, Rhen M, Hinton JC (2003). Unravelling the biology of macrophage infection by gene expression profiling of intracellular *Salmonella enterica*. Mol. Microbiol.

[98] Forstner KU, Vogel J, Sharma CM (2014). READemption-a tool for the computational analysis of deep-sequencing-based transcriptome data. Bioinformatics.

[99] Nicol JW, Helt GA, Blanchard SG, Raja A, Loraine AE (2009). The Integrated Genome Browser: free software for distribution and exploration of genome-scale datasets. Bioinformatics.

[100] Love MI, Huber W, Anders S (2014). Moderated estimation of fold change and dispersion for RNA-seq data with DESeq2. Genome Biol..

[101] Thompson JD, Higgins DG, Gibson TJ, CLUSTAL W (1994). improving the sensitivity of progressive multiple sequence alignment through sequence weighting, position-specific gap penalties and weight matrix choice. Nucleic Acids Res..

[102] Crooks GE, Hon G, Chandonia JM, Brenner SE (2004). WebLogo: a sequence logo generator. Genome Res..

[103] Wheeler TJ, Eddy S (2013). R. nhmmer: DNA homology search with profile HMMs. Bioinformatics.

[104] Lindgreen S (2014). Robust identification of noncoding RNA from transcriptomes requires phylogenetically-informed sampling. PLoS Comput Biol..

[105] Griffiths-Jones S (2005). RALEE-RNA ALignment Editor in Emacs. Bioinformatics.

[106] Sittka, A., Pfeiffer, V., Tedin, K. & Vogel, J. The RNA chaperone Hfq is essential for the virulence of *Salmonella typhimurium*. *Mol. Microbiol*. **63**, 193–217 (2007). 10.1111/j.1365-2958.2006.05489.x PMC1810395 17163975

[107] Schneider CA, Rasband WS, Eliceiri KW (2012). NIH Image to ImageJ: 25 years of image analysis. Nat. Methods.

[108] Sharma CM, Darfeuille F, Plantinga TH, Vogel J (2007). A small RNA regulates multiple ABC transporter mRNAs by targeting C/A-rich elements inside and upstream of ribosome-binding sites. Genes Dev..

[109] Pernitzsch SR, Tirier SM, Beier D, Sharma CM (2014). A variable homopolymeric G-repeat defines small RNA-mediated posttranscriptional regulation of a chemotaxis receptor in *Helicobacter pylori*. Proc. Natl Acad. Sci. USA.

[110] Hipp. R, et. al. SQLite v3.22.0 (SQLite Development Team, 2015).

[111] Carver T, Thomson N, Bleasby A, Berriman M, Parkhill J (2009). DNAPlotter: circular and linear interactive genome visualization. Bioinformatics.

