# Supplementary material for: A high-resolution transcriptome map identifies small RNA regulation of metabolism in the gut microbe Bacteroides thetaiotaomicron
Source: Nat Commun. 2020 Jul 16;11:3557. doi: 10.1038/s41467-020-17348-5 (PMC7366714; doi:10.1038/s41467-020-17348-5)
Supplement: Supplementary file 1 — Supplementary Information [file 41467_2020_17348_MOESM1_ESM.pdf]

Supplementary Information

A high-resolution transcriptome map identifies small RNA regulation of metabolism in the gut microbe *Bacteroides thetaiotaomicron*

Ryan et al.

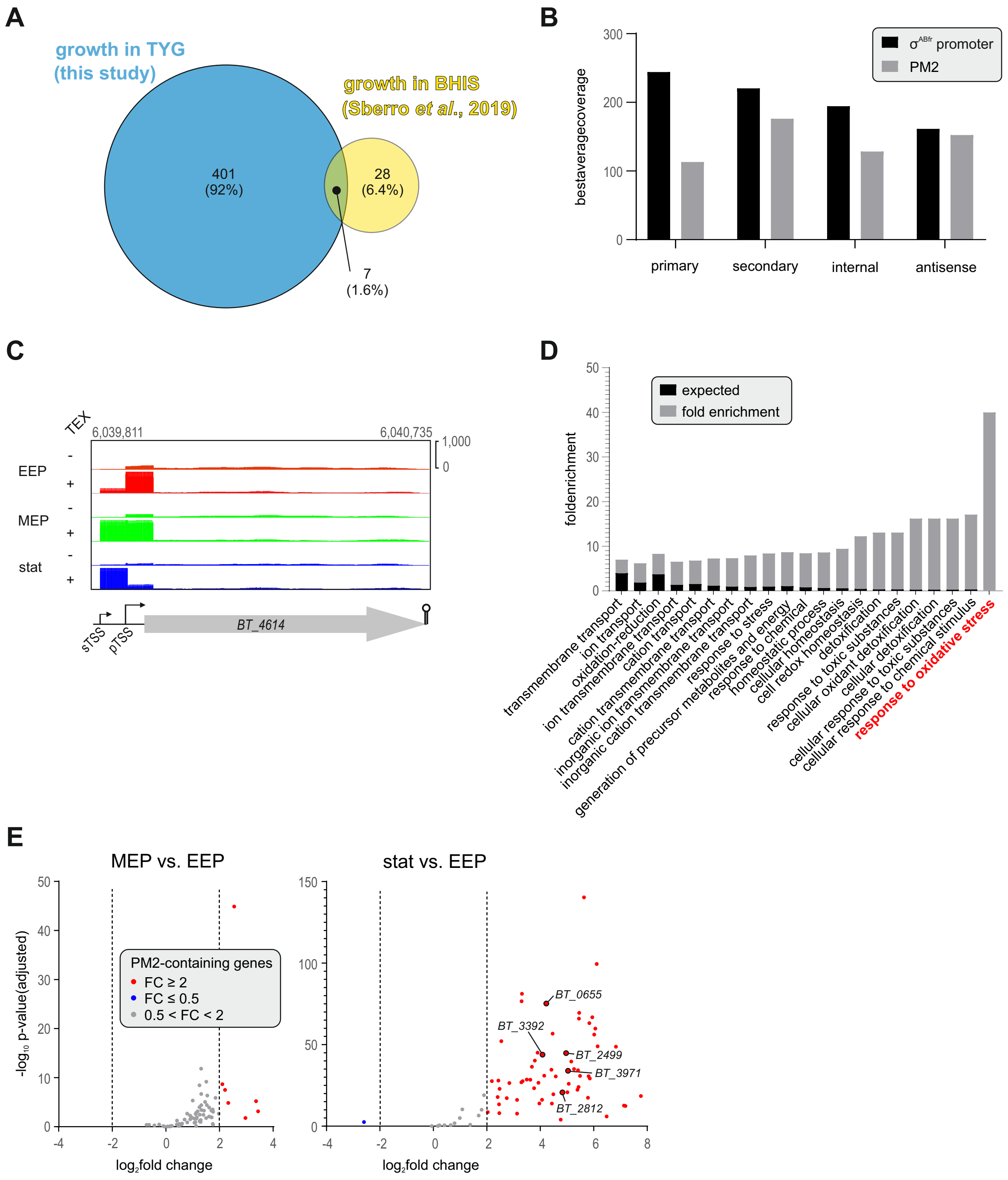

A
growth in TYG
(this study)
growth in BHIS
(Sberro et al., 2019)
401
(92%)
28
(6.4%)
7
(1.6%)
B
σ^ABfr promoter
PM2
bestaveragecoverage
300
200
100
0
primary
secondary
internal
antisense
C
TEX
6,039,811
6,040,735
1,000
0
EEP
MEP
stat
sTSS
pTSS
BT_4614
D
expected
fold enrichment
foldenrichment
50
40
30
20
10
0
transmembrane transport
ion transport
oxidation-reduction
ion transmembrane transport
cation transport
cation transmembrane transport
inorganic ion transmembrane transport
inorganic cation transmembrane transport
generation of precursor metabolites and energy
response to stress
response to chemical
homeostatic process
cellular homeostasis
cell redox homeostasis
detoxification
response to toxic substances
cellular oxidant detoxification
cellular response to toxic substances
cellular detoxification
cellular response to chemical stimulus
response to oxidative stress
E
MEP vs. EEP
stat vs. EEP
PM2-containing genes
FC ≥ 2
FC ≤ 0.5
0.5 < FC < 2
-log10 p-value(adjusted)
log2fold change
BT_0655
BT_3392
BT_2499
BT_3971
BT_2812

Supplementary Figure 1: Small protein candidates and inspection of promoter motif 2.

A: Venn diagram depicting the overlap of the small open reading frame (sORF) candidates predicted by ANNOgesic in the current study (growth for 4, 7, and 10 h in TYG; blue) or by ribosome profiling in [2] (growth for 72 h in BHIS; yellow). **B:** Bar graph illustrating the average read coverage of transcripts initiated by TSSs associated with promoter motif 2 (PM2) in comparison to genes with the canonical σ^{ABfr} promoter. **C:** Coverage plot over *BT_4614* (encoding a hypothetical protein) shows the growth phase-dependent transition from the σ^{ABfr} promoter-associated primary TSS (pTSS) in early exponential phase (EEP; red) to the PM2-associated secondary TSS (sTSS) upon entry into mid-exponential phase (MEP; green). **D:** PANTHER overrepresentation test of PM2-associated genes reveals an enrichment of genes involved in the response to oxidative stress (expected fold enrichment by chance [black] = 0.13; actual fold enrichment [grey] = 39.85). **E:** Volcano plots of the expression of genes initiated by PM2-associated pTSSs between the indicated growth stages in TYG (considered were the '–TEX' samples; n = 3 per condition). Red dots denote a $\log_2$ fold change ≥2 and blue dots denote a $\log_2$ fold change ≤–2. Red genes labeled by name belong to the GO-term 'response to oxidative stress' (that was enriched in panel D). Source data are provided as a Source Data file.

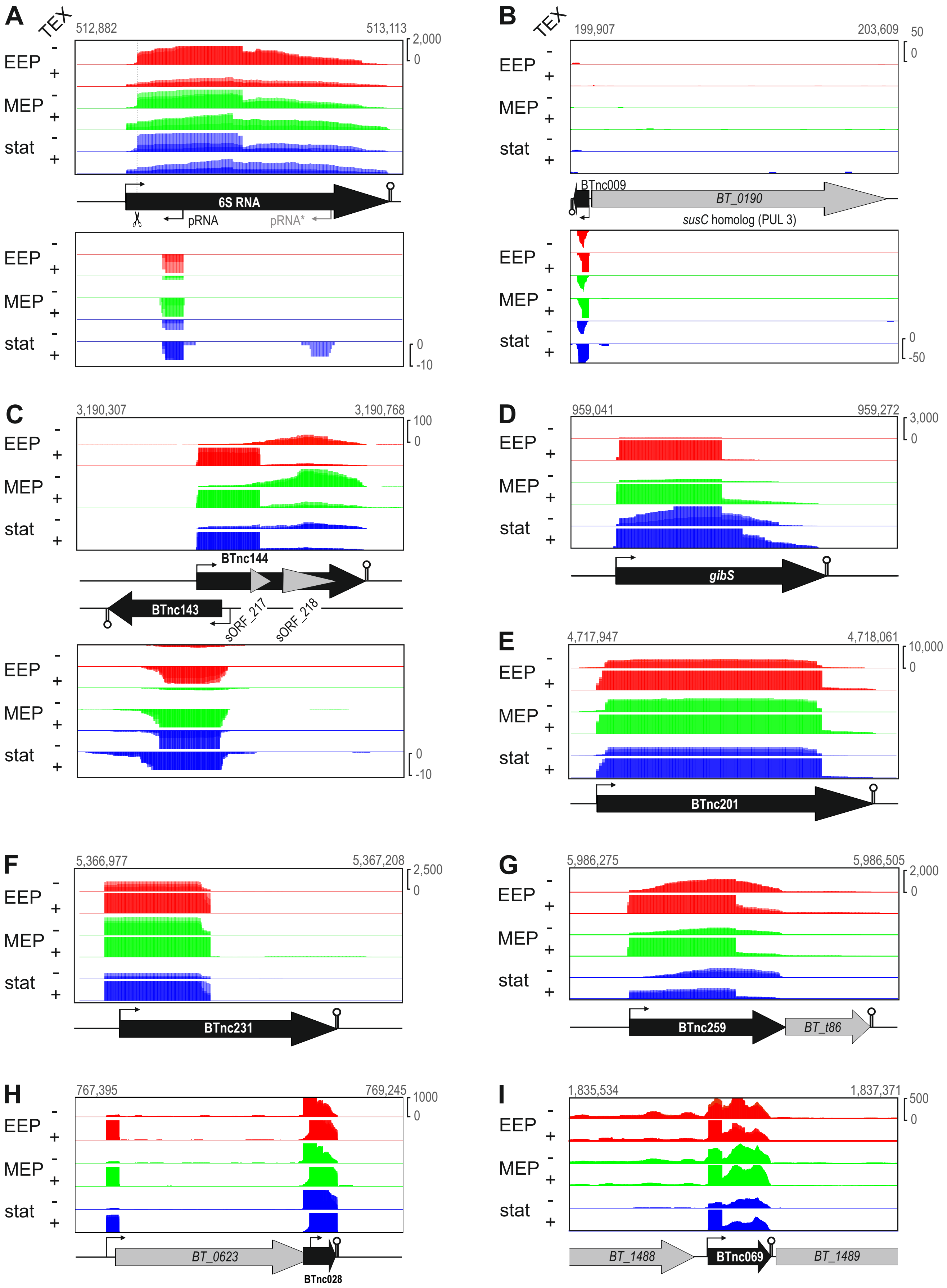
A
TEX
512,882
513,113
2,000
0
EEP
MEP
stat
6S RNA
pRNA
pRNA*
-10
B
199,907
203,609
50
BTnc009
BT_0190
susC homolog (PUL 3)
-50
C
3,190,307
3,190,768
100
BTnc144
BTnc143
sORF_217
sORF_218
D
959,041
959,272
3,000
gibS
E
4,717,947
4,718,061
10,000
BTnc201
F
5,366,977
5,367,208
2,500
BTnc231
G
5,986,275
5,986,505
BTnc259
BT_t86
H
767,395
769,245
1000
BT_0623
BTnc028
I
1,835,534
1,837,371
500
BT_1488
BTnc069
BT_1489

Supplementary Figure 2: Coverage plots of ncRNAs in *B. thetaiotaomicron*.

A-I: Mapped sequence reads from dRNA-seq ('+' refers to TEX treatment; '–' to no-TEX control) of *B. thetaiotaomicron* (AWS-001) during early exponential (EEP; red), mid-exponential (MEP; green) or stationary phase (stat; blue). For each condition, one out of three biological replicates is depicted. **A:** Housekeeping 6S RNA. Note the antisense-encoded pRNA and pRNA*. Using the default settings, ANNOgesic called a TSS for pRNA, but not for pRNA*, therefore the latter was drawn in grey. The scissors symbol refers to a processing site called be ANNOgesic. **B:** BTnc009, a PUL-associated *cis*-antisense RNA. While BTnc009 is constitutively expressed, the associated *susC* homolog was barely detected. **C:** BTnc143/-144 has a type-I toxin/antitoxin-like genomic organization. Two putative sORFs (sORF_217, -218) were predicted within BTnc144. **D-F:** GibS, BTnc231, and BTnc201 are intergenic sRNAs. **G:** 5'-derived sRNA candidate BTnc259. **H:** 3'-derived sRNA candidate BTnc028. **I:** BTnc069, an intra-operonic sRNA candidate. Bent arrows indicate TSSs and lollipop structures Rho-independent terminators identified in this study.

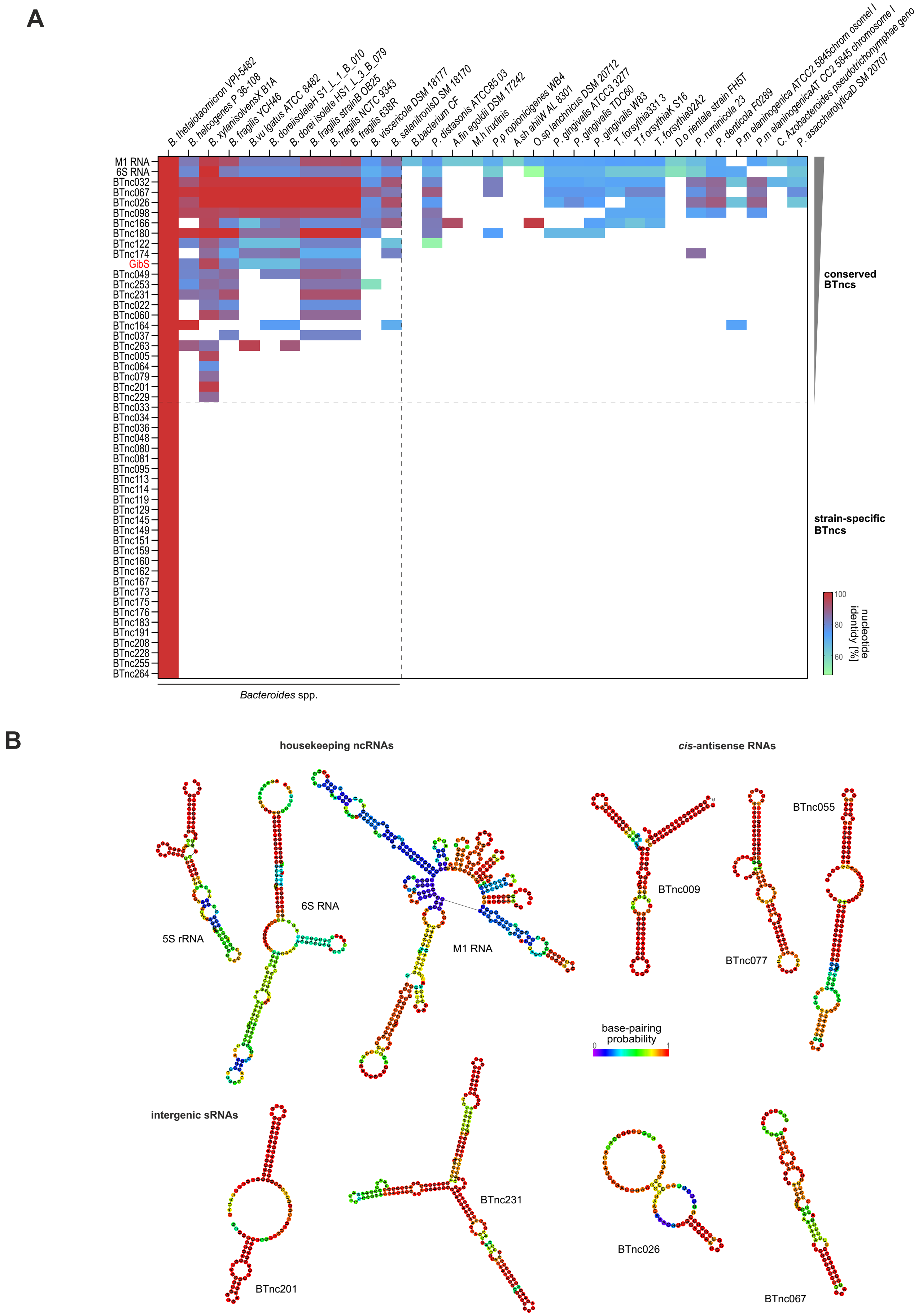

A
B. thetaiotaomicron VPI-5482
B. helcogenes P 36-108
B. xylanisolvensX B1A
B. fragilis YCH46
B.vu lgatus ATCC 8482
B. doreiisolateH S1_L_1_B_010
B. dorei isolate HS1_L_3_B_079
B. fragilis strainB OB25
B. fragilis NCTC 9343
B. fragilis 638R
B. viscericola DSM 18177
B. salanitronisD SM 18170
B.bacterium CF
P. distasonis ATCC85 03
A.fin egoldii DSM 17242
M.h irudinis
P.p ropionicigenes WB4
A.sh ahiiW AL 8301
O.sp lanchnicus DSM 20712
P. gingivalis ATCC3 3277
P. gingivalis TDC60
P. gingivalis W83
T. forsythia331 3
T.f orsythiaK S16
T. forsythia92A2
D.o rientale strain FH5T
P. ruminicola 23
P. denticola F0289
P.m elaninogenica ATCC2 5845chrom osomeI I
P.m elaninogenicaAT CC2 5845 chromosome I
C. Azobacteroides pseudotrichonymphae geno
P. asaccharolyticaD SM 20707
M1 RNA
6S RNA
BTnc032
BTnc067
BTnc026
BTnc098
BTnc166
BTnc180
BTnc122
BTnc174
GibS
BTnc049
BTnc253
BTnc231
BTnc022
BTnc060
BTnc164
BTnc037
BTnc263
BTnc005
BTnc064
BTnc079
BTnc201
BTnc229
BTnc033
BTnc034
BTnc036
BTnc048
BTnc080
BTnc081
BTnc095
BTnc113
BTnc114
BTnc119
BTnc129
BTnc145
BTnc149
BTnc151
BTnc159
BTnc160
BTnc162
BTnc167
BTnc173
BTnc175
BTnc176
BTnc183
BTnc191
BTnc208
BTnc228
BTnc255
BTnc264
conserved BTncs
strain-specific BTncs
100
80
60
nucleotide identity [%]
Bacteroides spp.
B
housekeeping ncRNAs
cis-antisense RNAs
5S rRNA
6S RNA
M1 RNA
BTnc009
BTnc055
BTnc077
base-pairing probability
0
1
intergenic sRNAs
BTnc201
BTnc231
BTnc026
BTnc067

Supplementary Figure 3: Conservation and secondary structure of ncRNAs in *B. thetaiotaomicron*.

A: Conservation of high-confidence intergenic sRNAs (n = 49) in Bacteroidia using nhmmer and WAR (see materials and methods). Percent sequence identities relative to reference sequences from *B. thetaiotaomicron* VPI-5482 are indicated by color. **B:** Secondary structure predictions of representative ncRNAs using RNAfold [1] at default settings. The color scale indicates the probability of base pairing.

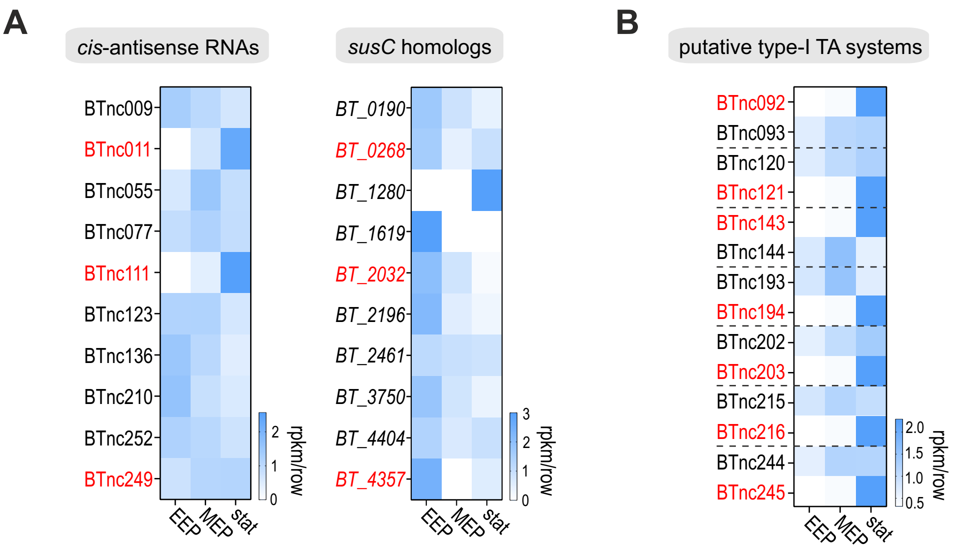

Supplementary Figure 4: Expression pattern of PUL-associated *cis*-antisense RNA candidates and putative type-I TA systems.

A: Heatmap shows the RNA-seq data of putative PUL-overlapping *cis*-antisense RNAs (left) and of their cognate *susC* homologs (right; in the same order) over growth in TYG medium. Relative expression refers to the rpkm divided by the row average of the respective genes in wild-type *B. thetaiotaomicron* (AWS-001) in early exponential (EEP), mid-exponential (MEP), and stationary phase as deduced from three biological replicates of the respective '–TEX' samples. PUL-associated *cis*-antisense RNAs, whose expression anti-correlated with that of their corresponding *susC* homolog, are highlighted in red. **B:** Heatmap displays expression data of putative type-I toxin-antitoxin (TA) systems, analogous to panel A. All TA systems show a similar expression pattern with the presumed toxin mRNAs (black) expressed at constant – albeit low – levels and the putative antitoxin (red) specifically induced in stationary phase.

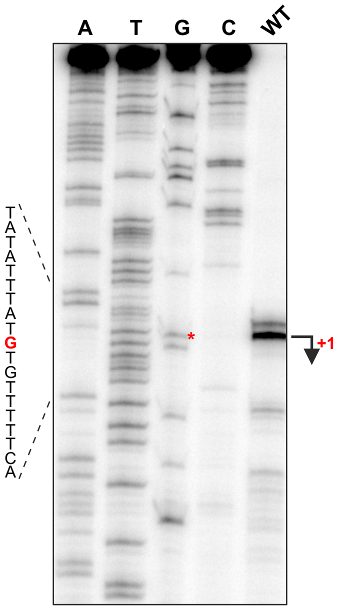

Supplementary Figure 5: Primer-extension confirms the 5' end of GibS.

Total RNA was isolated from *B. thetaiotaomicron* (WT; AWS-001) grown to stationary phase in TYG medium (n = 2 independent experiments). 10 µg of DNase I-treated RNA were used for primer-extension analysis with 5' end-labeled AWO-348. A sequencing ladder (A, T, G, C) ranging from 50 nt upstream to 87 nt downstream of the GibS TSS was generated using oligonucleotides AWO-347/-348. The primary TSS detected by primer-extension (red 'G') coincides with the TSS predicted by dRNAseq. Additionally, there is a second, less frequent TSS (the 'T' right upstream of the primary TSS), which seems to be also used *in vivo* (see Fig. 4B).

A

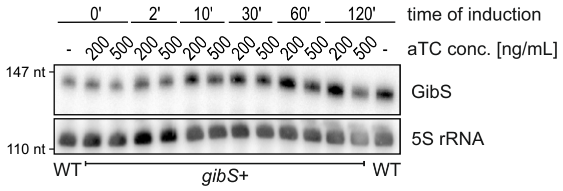

B

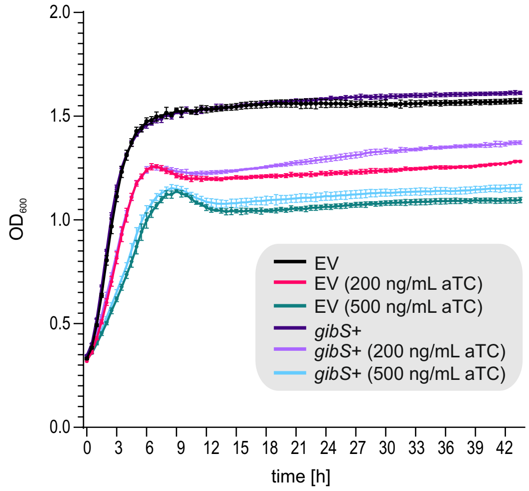

Supplementary Figure 6: Dosage and temporal effects of anhydrotetracycline on GibS induction and bacterial growth.

A: Northern blot of GibS expression in *B. thetaiotaomicron* wild-type (AWS-001) and strain *gibS*+ (AWS-035) grown in presence of the indicated concentrations of anhydrotetracycline (aTC) and for the indicated time periods in TYG medium. 5S rRNA was the loading control. **B:** Effect of different aTC concentrations on the growth kinetics of strain *gibS*+ and the parental strain harboring an empty vector control (EV; AWS-058). The mean growth curves in TYG medium supplemented with aTC as indicated are shown and error bars refer to +/– standard deviation from three biological replicates. Source data are provided as a Source Data file.

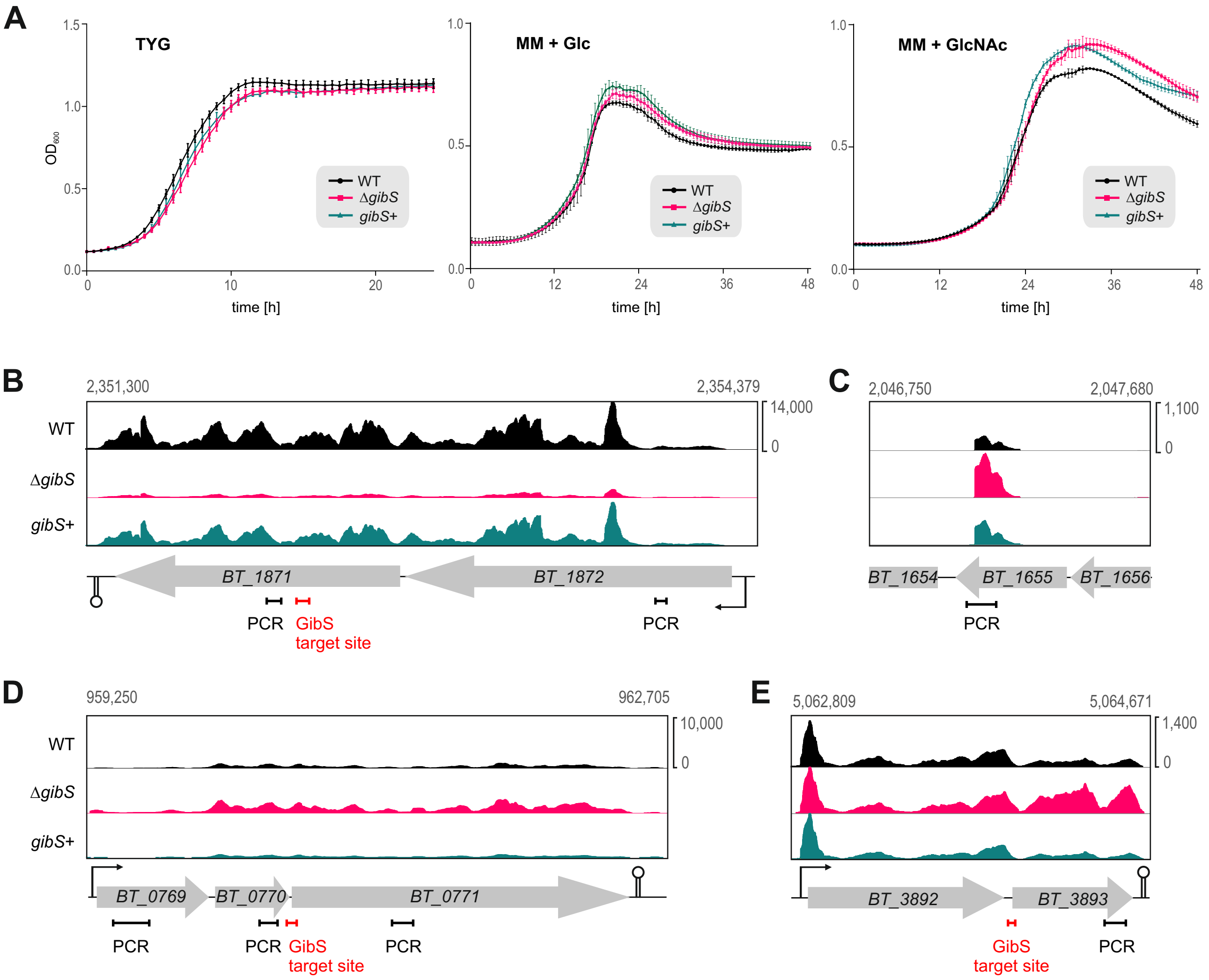

Supplementary Figure 7: Characterization of Δ*gibS* and *gibS*+ mutant strains and expression profiles of GibS target candidates.

A: Growth curves of the isogenic wild-type *B. thetaiotaomicron* (AWS-003), a deletion mutant of *gibS* (Δ*gibS*; AWS-028), and a complemented strain (*gibS*+; AWS-035) in TYG or minimal medium (MM) supplemented with either glucose (Glc) or N-acetyl-D-glucosamine (GlcNAc). Data refer to the mean +/− standard deviation from three biological replicates grown under anaerobic conditions over a period of 24 h (TYG) or 48 h (MM). **B-E:** Read coverage plots of *BT-1871-72* (B), *BT_1655* (C), *BT0769-71* (D), and *BT_3892-93* (E) in the Δ*gibS* mutant relative to the wild-type and *gibS*+ strains. Bent arrows denote the identified TSSs and lollipop structures the predicted Rho-independent terminators. The GibS targeting regions (see Fig. 5C) are marked in red and qRT-PCR amplicons in black. Read coverage for one representative out of two biological replicate samples per strain is depicted. Source data are provided as a Source Data file.

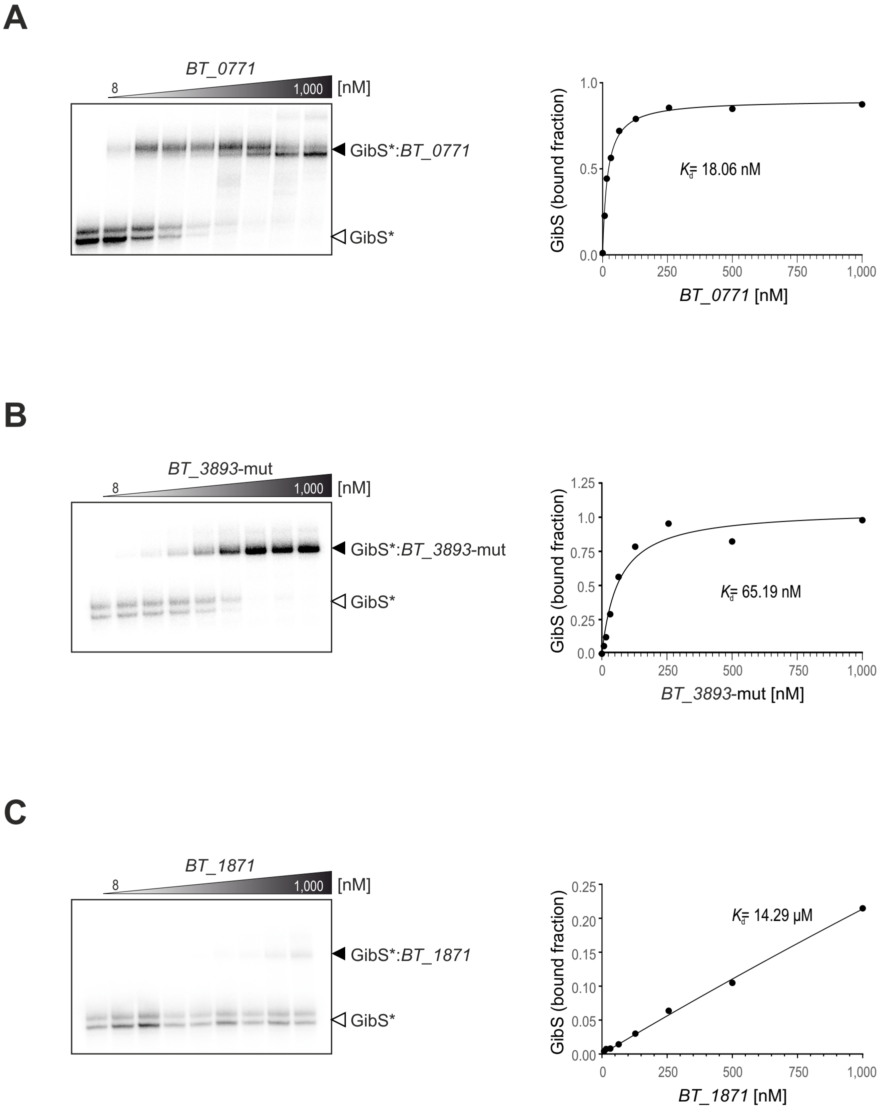

Supplementary Figure 8: Electrophoretic mobility shift assays of GibS and putative target mRNAs.

Gel-shift assays with 4 nM of 5' end-labeled GibS sRNA in the presence of increasing concentrations of an unlabeled segment of the translation initiation region of *BT_0771* (**A**), a mutated variant of *BT_3893* ('mut') (**B**), or a segment of the *BT_1871* coding sequence (**C**) that include the *in silico*-predicted regions of partial complementarity to GibS (see Fig. 5C). Left: Representative gel images. The positions of free and bound GibS are indicated to the right of the gels. Right: Quantification of binding affinities. The data refers to the mean of two independent replicate experiments, from which the equilibrium dissociation constants (K_d) were inferred. Source data are provided as a Source Data file.

REFERENCES

1 Lorenz, R. *et al.* ViennaRNA Package 2.0. *Algorithms for molecular biology : AMB* **6**, 26, doi:10.1186/1748-7188-6-26 (2011).

2 Sberro, H. *et al.* Large-Scale Analyses of Human Microbiomes Reveal Thousands of Small, Novel Genes. *Cell* **178**, 1245-1259 e1214, doi:10.1016/j.cell.2019.07.016 (2019)
