# Supplementary material for: A high-resolution transcriptome map identifies small RNA regulation of metabolism in the gut microbe Bacteroides thetaiotaomicron
Source: Nat Commun. 2020 Jul 16;11:3557. doi: 10.1038/s41467-020-17348-5 (PMC7366714; doi:10.1038/s41467-020-17348-5)
Supplement: Supplementary file 2 — Description of Additional Supplementary Files [file 41467_2020_17348_MOESM2_ESM.docx]

Description of Additional Supplementary Files

**Supplementary Data 1:** Transcription start sites in the genome of B. thetaiotaomicron VPI-5482 as identified in the differential RNA-seq screen. The table contains information on TSS prediction statistics as well as ANNOgesic annotations of TSSs, sorted by class. TSSs that are detected or enriched are denoted by a “1” alongside the specific condition, while those that are not detected are denoted by “0”. Note that a TSS can belong to more than one class. Red highlighted rows indicate TSS assignments that were rejected after manual curation. (XLSX)

**Supplementary Data 2:** List of identified noncoding RNA candidates from diverse classes. The table lists annotated noncoding RNAs that are grouped by category with additional information on putative sRNA duplications. (XLSX)

**Supplementary Data 3:** List of predicted small open reading frame candidates. The table lists ANNOgesic annotations of putative sORFs. (XLSX)

**Supplementary Data 4:** DNA oligonucleotides, plasmids, and bacterial strains used in this study. The table lists information pertaining to the DNA oligonucleotides, plasmids, and bacterial strains that were obtained or generated and used in this study. (XLSX)

**Supplementary Data 5:** RNA-seq read alignment statistics. The table contains read mapping statistics for the dRNA-seq analysis (Figs. 1-3) as well as the comparative transcriptomics of the wild-type, ∆gibS, and gibS+ strains (Fig. 5A). (XLSX)

**Supplementary Data 6:** Rho-independent terminators predicted by differential RNAseq and ANNOgesic. The table lists putative intrinsic terminators that were assigned by the ANNOgesic pipeline along with information on whether a corresponding read coverage decrease was detected. (XLSX)
